# Paper‐Based Electrodes for Flexible Energy Storage Devices

**DOI:** 10.1002/advs.201700107

**Published:** 2017-05-29

**Authors:** Bin Yao, Jing Zhang, Tianyi Kou, Yu Song, Tianyu Liu, Yat Li

**Affiliations:** ^1^ Department of Chemistry and Biochemistry University of California Santa Cruz California 95064 United States

**Keywords:** batteries, electrodes, flexible, paper, supercapacitors

## Abstract

Paper‐based materials are emerging as a new category of advanced electrodes for flexible energy storage devices, including supercapacitors, Li‐ion batteries, Li‐S batteries, Li‐oxygen batteries. This review summarizes recent advances in the synthesis of paper‐based electrodes, including paper‐supported electrodes and paper‐like electrodes. Their structural features, electrochemical performances and implementation as electrodes for flexible energy storage devices including supercapacitors and batteries are highlighted and compared. Finally, we also discuss the challenges and opportunity of paper‐based electrodes and energy storage devices.

## 1 Introduction

Flexible electronics have attracted extensive attention due to their potential for future hand‐held, potable consumer and wearable electronics. Electronic or optoelectronic components on flexible substrates enable novel applications, such as flexible display, electronic textile, artificial electronic skin, distributed sensors, etc.^1^, ^2^, ^3^, ^4^, ^5^, ^6^, ^7^, ^8^, ^9^ Whilst various flexible electronics are commercially available nowadays, the development of flexible energy and power sources for these devices is slow.^10^, ^11^ Such a “rate‐limiting step” has greatly impeded the commercialization of these electronics. In order to overcome this limiting factor, extensive efforts have been devoted to make flexible and high performance energy storage devices.^12^, ^13^, ^14^, ^15^, ^16^, ^17^, ^18^, ^19^

Among all flexible energy storage devices, supercapacitors and Li‐based batteries (e.g., Li‐ion, Li‐S and Li‐O_2_ batteries) stand out because of their ease of fabrication, compatibility with other electronic devices and excellent electrochemical performance.^17^, ^20^, ^21^, ^22^, ^23^, ^24^ They are typically composed of two electrodes (cathode and anode), separator, current collector, electrolyte and packing materials.^20^, ^25^, ^26^, ^27^ The two electrodes are the essential parts that render high flexibility of the devices. Traditionally, metal foils (e.g., Cu, Al and Ti foils)^28^, ^29^, ^30^ and certain plastics (e.g., polyethylene terephthalate)^31^ are used as substrates for making flexible electrodes. However, these substrates have obvious drawbacks. The large mass densities of electrochemically inactive metals considerably increase the total device weight. Additionally, they can be readily oxidized and cause significant drop of electrical conductivity. For the flexible plastic substrates, the weak affinity between active materials and plastic substrates often leads to the detachment of active materials during the electrode deformation induced by cycling, which greatly limits their durability.^28^, ^29^, ^30^, ^31^ Therefore, development of novel and reliable flexible electrodes is urgent and highly desirable.^12^, ^13^, ^14^, ^32^, ^33^

Paper, as one of the most ancient flexible products invented 2000 years ago and is still used today for information recording and packing, is one of the promising alternatives to conventional flexible substrates.^30^ Since 1960s, paper has been researched and employed as substrates for a diverse array of electronics, namely paper electronics,^34^ including displays,^35^ sensors,^36^, ^37^ transistors,^38^ radiofrequency identification devices,^39^ generators,^40^, ^41^ light‐emitting diodes,^42^ etc. Papers are promising electrode materials because of their wide availability, low cost, light weight, environmental friendliness, recyclability and bendability.^14^, ^34^, ^43^, ^44^, ^45^, ^46^ Typical paper electrodes are made by depositing or growing active materials onto traditional papers. But due to their poor electrical conductivities (10^11^–10^15^ Ω sq^−1^),^34^ additional chemical modifications (e.g., coating conductive layers for example) are required to make them electrically conductive.^12^, ^14^, ^47^, ^48^

Alternatively, a variety of other electrically conductive materials have been assembled into paper‐like film structures.^49^, ^50^, ^51^ These materials, mainly including carbon‐based materials such as carbon nanotube, graphene, and carbon fibers, can be directly fabricated into self‐supporting paper‐like electrodes. Paper‐like electrodes not only inherit the superior flexibility from paper‐supported electrodes, but also exhibit outstanding electrochemical performances as the electrode materials are electrochemically active. In light of the ultra‐small electrical resistance and volume, these paper‐like electrodes are promising materials that could achieve ultra‐high power density and energy density simultaneously.

In this review, we will present the recent advances in synthesis, characterization and applications of paper‐based electrodes, including both paper‐supported electrodes and paper‐like electrodes. Specifically, we will begin with the introduction on research progress of paper‐supported electrodes (Section 2), which are fabricated by applying active materials onto cellulose papers. Then paper‐like electrodes will be discussed in Section 3, including graphene‐based, carbon nanotubes‐based, carbon fiber‐based and carbon‐free paper‐like electrodes, which are fabricated via the controlled assembly of active materials into free‐standing film structures. Section 4 will focus on the applications of these paper‐based electrodes for diverse energy storage devices including supercapacitors, Li‐ion batteries, Li‐S batteries and Li‐O_2_ batteries with an emphasis on the first two devices. Conclusions, comments and outlooks will be given in the last section (Section 5).

## 2 Paper‐Supported Electrodes

Paper, which is made of randomly interconnected cellulose fibers, is a product after a three‐step treatment of wood cellulose pulp suspension: dewatering, pressing and heating. Mineral fillers, such as calcium carbonate, chalk and clay, are usually mixed with the cellulose pulps to increase smoothness. To enhance the brightness of paper, fluorescent whitening agents (e.g. stilbenes) are added in the process of fabrication. The mechanical properties of paper can be readily tuned by adjusting the length, diameter and physical and chemical nature of cellulose fibers used for production. In general, papers made of longer and wider fibers are more robust than counterparts composed of shorter and thinner fibers.^34^ Various types of papers with different purposes (daily‐use office photocopying paper, weighting paper, filter paper, Kimwipes paper etc.) are widely used in daily lives. Besides mechanical properties, paper's optical properties can also be changed. It has been reported that by filling inter‐fiber space using transparent materials (e.g., wax) with reflective constant that is closed to the cellulose (≈1.5), it is able to fabricate transparent papers.^34^ Reducing the diameter of cellulose fibers from micro‐meters (≈20 µm) to nanometers (≈20 nm) will increase paper transparency.^52^, ^53^, ^54^

**Figure 1:**
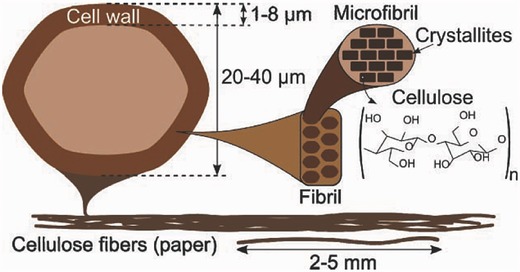
Schematic illustration showing the hierarchical structure of cellulose fibers. Reproduced with permission.^34^

Each paper fiber possesses a hierarchical structure as shown in **Figure** **1**. The surface layer of each fiber, which has a thickness of 1–8 µm, is made of small fibrils bundled together. Each fibril is composed of small microfibril bundles with a diameter between 3 and 20 nm. Two regions exist in every microfibril: the amorphous regions and the crystalline regions. Hemicellulose and lignin fill in the amorphous regions. In the crystalline regions, there are thousands of 5 µm long cellulose chains. Cellulose fibers interconnect and weave altogether to form the paper. The typical thickness and areal mass density of a paper substrate are ≈100 µm and 80 g m^−2^, respectively.^34^ Fibrils and microfibrils are held together by hydrogen bonding rendered by surface hydroxyl groups. These hydrogen bonds play a critical role in fabricating paper‐supported electrodes, by anchoring the guest materials onto paper surface.^12^, ^30^, ^47^, ^55^, ^56^, ^57^, ^58^

For making paper‐supported electrodes, pre‐treatments of paper substrates to eliminate inactive additives and increase porosity are needed. A typical procedure was reported by Yao et al.^14^: immerse a piece of printing paper into an aqueous solution containing 0.3 M hydrochloric acid (HCl) for about 10 min, then wash with deionized water thoroughly and let it dry at room temperature. Pre‐treatments usually lead to reduction on both weight and thickness due to the removal of mineral fillers and fluorescent whitening agents, and resulting in a more porous open structure that is often beneficial for paper‐supported electrodes.

### 2.1 Electrode Fabrication

**Figure 2:**
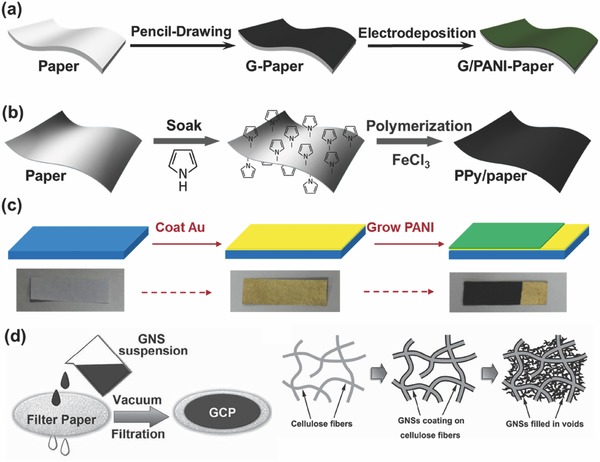
Schematic illustrations of four protocols for making paper‐supported electrodes. a) Graphite/polyaniline (PANI)‐Paper fabricated by Pencil‐drawing method. Reproduced with permission.^14^ Copyright 2013, Elsevier. b) Polypyrrole(PPy)‐coated paper fabricated by soaking and polymerization method. Reproduced with permission.^12^ Copyright 2013, Royal Society of Chemistry. c) PANI/Au/paper electrode fabricated by vaporization method. Reproduced with permission.^32^ d) graphene‐cellulose paper (GCP) electrode fabricated by vacuum filtration method. Reproduced with permission.^59^

**Figure 3:**
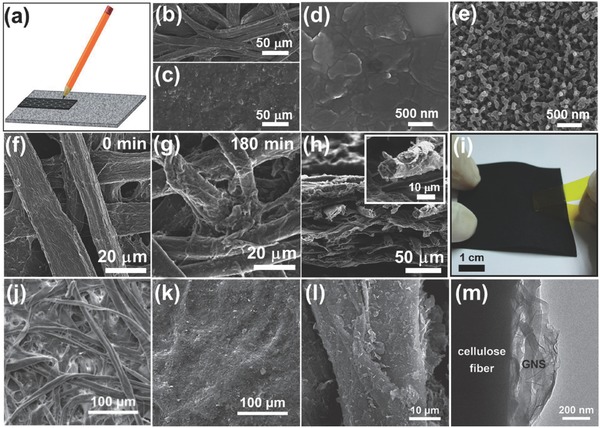
Microstructures of paper‐based electrodes. a) Schematic diagram of drawing conductive electrode on cellulose paper using a graphite pencil. Reproduced with permission.^48^ Copyright 2013, Royal Society of Chemistry. b–e) Scanning electron microscopy images of (b) blank A4 printing paper, (c, d) graphite paper, (e) G/PANI‐Paper with polyaniline deposition time of 120 min. Reproduced with permission.^14^ Copyright 2013, Elsevier. f–g) Scanning electron microscopy images for paper coated by polypyrrole with different polymerization times of 0 and 180 minutes, respectively. h) Cross‐section scanning electron microscopy image of the 180 minutes polypyrrole/paper composite. The inset shows an enlarged polypyrrole‐coated cellulose fiber. i) Polypyrrole‐coated paper passes the tape test shows that the strong mechanical property. Reproduced with permission.^12^ Copyright 2013, Royal Society of Chemistry. j,k) SEM images of the filter paper or GCP surfaces with different GNS loading amounts. j) 0 wt% (pristine filter paper), and k) 7.5 wt%. l) SEM and m) TEM images of a cellulose fiber in a GCP membrane showing GNSs anchored on the fiber surface. Reproduced with permission.^59^

**Figure 4:**
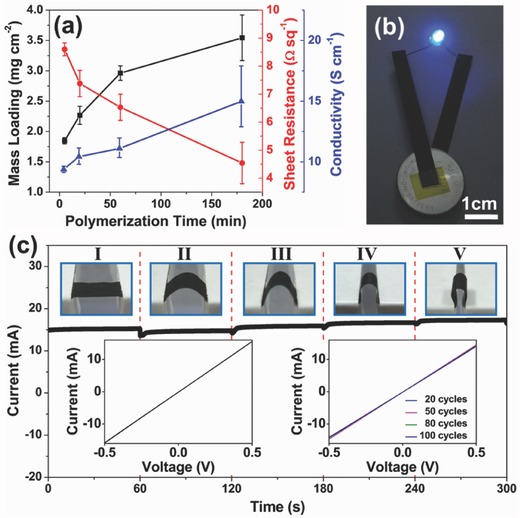
Electrical performance of paper electrode. a) Mass loadings of polypyrrole (▪), sheet resistance (•) and conductivity (▴) of the fabricated polypyrrole‐coated paper with respect to polymerization time of 5, 20, 60 and 180 minutes. b) Polypyrrole‐coated paper as electrical connections to light a blue LED by a homemade 2.7 V battery. c) Current‐time curves of polypyrrole‐coated paper bent with different curvatures under a constant voltage. The upper insets labeled I, II, III, IV and V reveal the five bending states. The lower left inset shows the current‐voltage curve of polypyrrole‐coated paper without bending. The lower right inset records the current‐voltage curves of polypyrrole‐coated paper after 20, 50, 80 and 100 cycles of bending. Reproduced with permission.^12^ Copyright 2013, Royal Society of Chemistry.

**Figure** **2** summarizes four main protocols of making paper‐supported electrodes. (1) Pencil‐drawing or printing method. A thin layer of graphite or other conductive materials is first deposited onto the paper surface, followed by electrodeposition of other active materials (Figure 2a); (2) Soaking and polymerization method. Paper is coated with conducting polymers by in situ polymerizations of monomers that are pre‐soaking paper substrates in monomer solutions (Figure 2b); (3) Vaporization method. A thin layer of metal is first deposited to make paper substrate more electrically conductive. Conducting polymers or metal oxides are subsequently electro‐deposited onto the metal coated paper substrates (Figure 2c); (4) Vacuum filtration method. Highly conductive and electrochemically active materials can be directly deposited onto a piece of filter paper through vacuum filtration. The guest materials can coat on cellulose fibers and fill the inter‐fiber voids (Figure 2d).

#### 2.1.1 Pencil Drawing

Pencil leads, the most accessible graphite products in our daily life, are composed of fine graphite particles held together by clay binders. Based on its hardness, pencils are classified on a scale from 9B to 9H.^60^ Their colors are different due to their different fraction of graphite and clay. When pencil traces are drawn on paper, the friction between pencil lead and paper rubs off graphite particles which in turn adhere to the paper fibers. Thus pencils can be viewed as a deployable form of ultrafine graphite particles. Pencil traces can be considered as conductive films made of percolated graphite particle network on paper. Pencil leads with different hardness can result in tunable areal conductivity. More specifically, drawing with 9B pencil lead could achieve highest conductive film, while using 9H pencil lead would come out the most inferior conductivity. These traces can be used to create any arbitrary shape and patterns and they are quite stable against chemicals, moisture and UV irradiation.^60^ This pencil‐based approach has been widely used in making electrodes for flexible energy storage devices recently.^14^, ^48^

Pencil‐drawing can also couple with other methods to make composite electrodes. Zheng et al. used a pure graphite rod to draw on Xerox cellulose paper to get a solvent‐free drawing electrode (**Figure** **3**a).^48^ A fairly low sheet resistance ≈233 Ω sq^−1^ was achieved by drawing in orthogonal direction that can deposit graphite more uniformly on the paper. An aqueous supercapacitor built via the assembly of two pencil‐drawing electrodes can deliver a high capacitance of 1.13 mF cm^−2^ and retain 90% its initial capacitance after 15000 cycles. Although the device shows stable performance, its areal capacitance is still relatively small. Later Yao et al. used an electrodeposition method to grow polyaniline (PANI) network on the pencil‐drawing graphite paper.^14^ The scanning electron microscopy (SEM) images showed pencil‐drawing process formed a uniform layer of graphite sheets on the paper surface, while three dimensional PANI networks were further grown on the graphite paper (Figure 3b–e). The PANI coated paper electrode achieved a higher areal capacitance of 355.6 mF cm^−2^. The flexible solid‐state supercapacitor fabricated by sandwiching polyvinyl alcohol (PVA)/H_2_SO_4_ gel electrolyte film between two PANI/graphite electrodes displayed light‐weight characteristic, with 219 µm in thickness and 30.8 mg in weight. Feng et al. further improved the performance by using two asymmetric paper electrodes.^61^, ^62^, ^63^ Graphite/Ni/Co_2_NiO_4_ paper electrode was fabricated by pencil drawing, followed by deposition of Ni and Co_2_NiO_4_ in sequence. Graphite/Ni/active carbon paper electrode and Graphite/Ni/Co_2_NiO_4_ paper were used as negative and positive electrode respectively to assemble an asymmetric supercapacitor, which achieve a high working voltage of 2V. It has an outstanding volumetric energy density of 2.48 mWh cm^−3^, which is much larger than the value obtained from other paper‐based symmetric devices.

#### 2.1.2 Printing

Printing on paper is a facile, fast and highly scalable method for making flexible paper electronics. A major challenge of using paper for flexible electronics is its rough surface, which generally needs planarization by surface treatment or surface coating before printing.^34^ However, this rough surface of paper is not problematic but advantageous for energy storage device such as supercapacitor and Li‐ion batteries.^30^ According to the different equipment used, printing can be classified to ink‐jet printing,^64^ Mayer rod printing,^30^ screen printing,^65^ gravure printing,^66^ etc. Some untraditional methods like using Chinese brush to deposit solution‐based materials on paper have also been demonstrated.^30^

Selecting and preparing the appropriate ink for printing is the key step in making printing‐based paper electrode. Hu et al. employed Mayer rod to coat uniformly dispersed CNT ink and Ag ink on ordinary office paper and successfully fabricate a CNT conductive paper.^30^, ^47^ This conductive paper reached a low sheet resistance around 10 Ω sq^−1^ for CNT coating and 1 Ω sq^−1^ for Ag nanowire coating. The CNT paper electrode achieved a specific capacitance of 200 F g^−1^, a specific energy of 30–47 Wh kg^−1^, a specific power of 200 kW kg^−1^ and a stable cycling life over 40000 cycles. This conductive CNT paper can also be used as a current collector that has been integrated with LiMn_2_O_4_ (cathode) and Li_4_Ti_5_O_12_ (anode) to assemble a full Li‐ion battery. The LiMn_2_O_4_ nanorod and Li_4_Ti_5_O_12_ nanopowder electrodes achieved initial discharge capacities of 110 mAh g^−1^ and 149 mAh g^−1^, and capacity retentions of 93% and 96% after 50 cycles, respectively.

To integrate anode, cathode and separator into a single sheet of paper and fabricate an integrated device, Hu et al. further demonstrated a high‐speed ink‐jet printing method to print single‐wall carbon nanotubes (SWCNT) on both sides of a pre‐treated polyvinylidene fluoride (PVDF) paper.^64^ The PVDF coating was employed to prevent CNT penetration through the micron‐sized pores in paper that can cause short circuit as all the components are integrated onto a single sheet of paper. A specific capacity of 33 F g^−1^ at a high specific power of 250 kW kg^−1^ was achieved in an organic electrolyte. Such a lightweight paper‐based supercapacitor is promising for powering paper electronics such as transistors or displays.

#### 2.1.3 Soaking and Polymerization

Conducting polymers, which were discovered in 1976, are one important type of electrode materials for energy storage due to their large capacitance, good electrical conductivity, ease of synthesis and low cost.^25^, ^32^, ^67^, ^68^, ^69^ Over the decades, conducting polymers have received significant attention in flexible energy storage devices. Among them, polypyrrole (PPy), polyaniline (PANI), poly[3,4‐ethtylenedioxythiophene] (PEDOT) and their derivatives have been widely investigated in supercapacitors due to their large specific capacitance, e.g., 480 F g^−1^ for PPy, 1284 F g^−1^ for PANI, and 210 F g^−1^ for PEDOT.^70^ As a 3D porous substrate, paper can be easily coated with conducting polymers due to its hydrogen bonding interaction with polymer monomers, which can then be polymerized into conducting polymers and form a 3D conducting network.

Yuan et al. simply soaked the daily printing paper into PPy monomer solution and then transferred into ferric chloride solution with hydrochloric acid to polymerize PPy onto the paper substrate. In this process, ferric chloride works as oxidation agent and hydrochloric acid acts as doping agent. After polymerization, the paper became black and conductive. The roughness of the cellulose fibers increased and the connections among fibers increased compared with the blank paper substrate (Figure 3f and 3g). More importantly, both the exterior and interior of cellulose fibers were coated with PPy uniformly (Figure 3h). Mechanical adhesion test showed that no obvious PPy could be observed on tape, demonstrating the good adhesion of PPy on the paper (Figure 3i). PPy coated paper electrode showed high gravimetric capacitance above 370 F g^−1^. The flexible solid‐state supercapacitor fabricated via sandwiching PVA/H_3_PO_4_ membrane both as electrolyte and separator displayed an areal capacitance of 0.42 F cm^−2^, a high energy density of 1 mWh cm^−3^ at a power density of 0.27 W cm^−3^ normalized to the device volume.

#### 2.1.4 Thermal Evaporation

Thermal evaporation is a commonly used method for thin film deposition. It involves two basic processes: vaporization of source material and condensation of vapor on a substrate. This evaporation process takes place in vacuum and usually it is directional. Paper is inherently not an ideal substrate for depositing continuous film, since its surface is uneven and rough. Besides, the interconnection between metal particles and paper substrate is relatively weak. However, this problem can be solved by coating a thin layer of polymer materials, such as PVA or parylene.^32^, ^71^

Yuan et al. prepared a PVA solution by mixing PVA powder with deionized water. Then, a piece of printing paper was immersed into the PVA solution until PVA fill the paper thoroughly. The treated paper was then air dried. A thin gold (Au) film (80 nm) was deposited on the PVA treated paper via E‐beam evaporation. This Au paper displayed a low sheet resistance of 7 Ω sq^−1^. PANI/Au paper electrodes were fabricated by electrodeposition of PANI nanowires on Au paper. The paper electrode showed a high specific capacitance of 560 F g^−1^, an areal capacitance of 0.8 F cm^−2^ and a high volumetric capacitance of 800 F cm^−3^ (based on the mass and thickness of active material).^32^

Wang et al. reported the first flexible micro‐supercapacitor on paper.^71^ They evaporated parylene onto a photographic paper for waterproofing and insulation purposes. 200 nm gold interdigital patterns were then directly thermally evaporated onto the parylene passivated paper using hard masks. Coral‐like polyaniline‐manganese oxide was then electrochemically deposited onto the interdigital gold electrode. Employing the PVA/H_3_PO_4_ gel electrolyte, this flexible solid‐state interdigital supercapacitor displayed a high areal capacitance of 94.73 mF cm^−2^ and a high areal energy density of 6.3 µWh cm^−2^.

#### 2.1.5 Vacuum Filtration

Vacuum filtration is one of the most popular methods in making paper‐supported electrodes because of its fast and scalable characteristics. Owing to its intrinsic 3D porous structure, paper can be used as a “filter” to block the materials with the size larger than its pore size and at the same time coat the surface of fibers and fill its pores with these materials. Vacuum filtration method has been used to prepare carbon based (e.g., graphene, graphite, CNT) paper‐supported electrodes.^58^, ^59^, ^72^, ^73^, ^74^, ^75^

Weng et al. fabricated the graphene paper by simply filtering a graphene nanosheets (GNSs) suspension through a filter paper.^59^ As seen from the SEM images in Figure 3j and 3k, the cellulose fiber surfaces and voids of the filter paper were covered and filled with GNSs. The strong binding between cellulose fibers and GNSs was ascribed to the strong electrostatic interaction between the functional groups on the fibers and the negatively charged GNSs. The resistivity of this graphene paper is 6 Ω·cm and only decrease 6% after being bent 1000 times. The graphene paper electrode displayed a gravimetric capacitance of 120 F g^−1^ (normalized to the mass of graphene) and retains more than 99% of its initial capacitance over 5000 cycles. Jabbour et al. manufacture flexible cellulose‐graphite paper electrode via direct infiltration of the hybrid slurry of graphite particles and cellulose, which resulted in a flexible free‐standing cellulose‐based graphite paper.^58^ This graphite paper showed excellent tensile properties with Young moduli ranging between 60 and 450 MPa. When the graphite paper was used as anode in Li‐ion batteries, it exhibited an excellent discharge capacity up to 350 mAh g^−1^, which is very close to the theoretical limit for graphite electrodes (i.e. 372 mAh g^−1^). The paper electrode also has stable performance over 120 cycles.

### 2.2 Optimizing Electrical Property of Paper Electrodes

Electrical conductivity is a critical factor that can greatly affect the electrochemical performance of paper electrodes. The resistance of the electrodes should be as low as possible. The total resistance includes the resistance of the active materials, resistance of the current collector, and interfacial resistance between active materials and current collector. Normally, the resistance of the current collector is relatively small. Thus, to decrease the resistance of active materials and interfacial resistance between electrode material and current collector is important for enhancing the electrode performance. A number of highly conductive materials such as conducting polymers,^12^, ^14^, ^25^, ^67^ carbon‐based materials,^76^, ^77^, ^78^, ^79^ metal nitrides,^80^, ^81^, ^82^ metal carbides^51^, ^83^ have been employed for fabricating electrodes. Moreover, various methods have been developed to improve the electrical conductivity of electrode materials with modest conductivity such as metal oxides.^84^, ^85^, ^86^ These approaches include doping,^87^, ^88^ introducing oxygen vacancy,^89^, ^90^ introducing low valence states^91^ and synthesizing hybrid electrodes with high conductive materials.^33^, ^92^

Some metrics can be used to evaluate the electric properties of paper‐based electrodes, such as sheet resistance, conductivity, and conductance stability under deformations, etc.^12^, ^14^ Yuan et al. successfully fabricated highly conductive PPy‐coated paper electrodes.^12^ The electrical conductivity of the PPy coated paper electrodes increased with increasing polymerization time and mass loading of PPy (**Figure** **4**a). Its sheet resistance can be as low as 4.5 Ω sq^−1^. The high conductivity of the PPy‐coated paper (15 S cm^−1^) assures that it can be used as a flexible lead to connect all the device components. As shown in Figure 4b, a homemade 2.7 V button battery light up a blue light‐emitting‐diode (LED) (the lowest operating voltage is 2.5 V) by using the PPy‐coated paper as a connecting lead. At a fixed voltage of 0.5 V, the PPy‐coated paper showed excellent conductance stability at different bending states, even at 180° (Figure 4c). Furthermore, the conductance of PPy‐coated paper remained almost constant after 100 cycles, indicating its high stability and flexibility, which is promising for the application in flexible electronics.

Although paper‐supported electrodes have only been developed in recent years, they have attracted much attention in the research community and shown great potential in flexible energy storage applications. In addition to the abovementioned examples, we also summarized the materials, synthesis method, and electrochemical performances of other paper‐supported electrodes and their applications in flexible energy storage devices in **Table** **1**.

**Table 1:** Performance of paper‐supported electrodes in flexible energy storage devices

Electrodes	Synthesis method	Device	Performance				Ref.
			Capacitance/Capacity	Power Density	Energy Density	Cycling Stability	
PPy‐paper	Soaking and polymerization	SC*	1.5 F cm^−2^	0.46 W cm^−3^	1 mWh cm^−3^	75.6% after 10000 cycles	12
			370 F g^−1^				
PPy‐bacterial cellulose paper	Vacuum filtration	SC	2.43 F cm^−2^	–	–	94.5% after 5000 cycles	74
PPy‐cladophora cellulose paper	Vacuum filtration	SC	315 C g^−1^	2.7 kW kg^−1^	1.75 Wh kg^−1^	No loss after 1500 cycles	72
PANI‐paper	Electrodeposition	SC	160 F g^−1^	–	–	81% after 1000 cycles	55
PANI‐graphite‐Paper	Pencil‐drawing Electrodeposition	SC	355.6 mF cm^−2^	0.054 W cm^−3^	0.32 mWh cm^−3^	83% after 10000 cycles	14
PANI‐nylon‐cellulose acetate‐paper	Electrospinning Electrodeposition	SC	402 F g^−1^	–	–	61% after 1000 cycles	93
PEDOT‐paper	In situ Polymerization	SC	145 F cm^−3^	1 W cm^−3^	1 mWh cm^−3^	91% after 2500 cycles	56
			115 F g^−1^				
PEDOT‐paper	Spin coating	SC	994 μF cm^−2^ *	64.70 W cm^−3^	1.77 mWh cm^−3^	No loss after 12000 cycles	94
			16.6 F cm^−3^ *				
CNT‐paper	Dip and dry	SC	115.83 F g^−1^	–	48.86 Wh kg^−1^	–	57
CNT‐paper	Mayer rod printing	SC	200 F g^−1^ *	200 kW kg^−1^	47 Wh kg^−1^	97% after 40000 cycles	47
CNT‐PVDF‐paper	printing	SC	33 F g^−1^	250 kW kg^−1^	–	96.9% after 2500 cycles	64
MnO_2_‐CNT‐paper	Dip and dry	SC	1162 F g^−1^	4 mW cm^−2^	4.2 µWh cm^−2^	97.8% after 20000 cycles	95
Si‐CNT‐paper	Vacuum filtration PECVD*	LIB*	2100 mAh g^−1^	–	–	77% after 100 cycles	96
PEDOT‐CNT‐paper	Ink‐jet printing	SC	23.6 F cm^−3^	89.1 mW cm^−3^	42 mWh cm^−3^	92% after 10000 cycles	97
h‐MnO_2_‐CNT‐paper	Printing	SC	1035 F g^−1^	81 kW kg^−1^	25.3 Wh kg^−1^	98.9% after 10000 cycles	98
			91.7 mF cm^−2^				
CNT‐MnO_2_‐CNT‐paper	Dip‐and‐dry electrodeposition	SC	327 F g^−1^	–	–	85% after 50000 cycles	99
LiMn_2_O_4_‐CNT‐paper	Mayer rods printing Pressing	LIB	110 mAh g^−1^	–	–	93% after 50 cycles	30
Li_4_Ti_5_O_12_‐CNT‐paper		LIB	149 mAh g^−1^	–	–	96% after 50 cycles	
Graphene‐paper	Vacuum filtration	SC	252 F g^−1^	2.4 kW kg^−1^	237 Wh kg^−1^	–	59
		LIB	540 mAh g^−1^	–	–	22.9% after 45 cycles	75
Graphite‐paper	Pencil‐drawing	SC	2.3 mF cm^−2^	35 kW kg^−1^	1.46 Wh kg^−1^	90% after 15000 cycles	48
Graphite‐paper	Vacuum filtration	LIB	350 mAh g^−1^	–	–	94.5% after 120 cycles	58
MnO_2_‐Ni‐paper	Electroless plating Electrodeposition	SC	1.9 F cm^−2^	2.5 mW cm^−2^	0.8 mWh cm^−3^	85.1% after 1000 cycles	100
MnO_2_‐Ni‐graphite‐paper	Pencil‐drawing Electrodeposition	SC	175 mF cm^−2^	48 mW cm^−3^	106 mWh cm^−3^	96% after 6000 cycles	62
				60 kW kg^−1^	66.2 Wh kg^−1^		
Mn_3_O_4_‐Ni‐graphite‐paper	Pencil‐drawing Electrodeposition	SC	432 mF cm^−2^	33 mW cm^−3^	0.35 mWh cm^−3^	83% after 12000 cycles	63
			432 F g^−1^				
Co_2_NiO_4_‐Ni‐graphite‐paper	Pencil‐drawing Electrodeposition	SC	734 mF cm^−2^	0.79 W cm^−3^	2.48 mWh cm^−3^	97.6% after 15000 cycles	61
				25.6 kW kg^−1^	80 Wh kg^−1^		
PANI‐Au‐paper	Evaporation and Electrodeposition	SC	560 F g^−1^	3 W cm^−3^	0.01 Wh cm^−3^	103% after 10000 cycles	32
			0.8 F cm^−2^				
Si‐CNTs‐CNC film	Vacuum filtration	LIB	3200 mAh g^−1^	–	–	57.5% after 100 cycles	101

*Notes: Supercapacitor (SC), Lithium‐ion batteries (LIB), Plasma‐enhanced chemical vapor deposition (PECVD).

## 3 Paper‐Like Electrodes

Most of the electrodes for energy storage devices are generally made by mixing particulate active materials with polymeric binders e.g., polyvinylidene fluoride (PVDF) and polytetrafluoroethylene (PTFE) and conducting agents (e.g. carbon black) with the help of appropriate solvent. The binders are used to glue the active materials and conducting agents as well as the current collector, whereas the conductive agent network helps to transfer electrons from active materials to current collector. Although this process is widely adopted in industry, the introduction of insulated binders increases both the contact resistance between particles as well as the resistance of the electrodes. Besides, the binders and additive conductive agents make up about 20–40% of the total electrode mass, which are seen as ‘dead mass' because they do not contribute to charge storage, but instead decrease the energy density of both the electrodes and devices.^102^

Flexible cellulose substrates can be used to support conductive active materials to achieve high performance in many flexible energy storage systems. However, the addition of electrochemically inactive cellulose paper substrate considerably lower the energy density and power density of supercapacitor device when they are normalized to the total electrode mass/volume. Thus, to eliminate the inactive paper substrate and fabricate paper‐like electrodes with active charge storage materials can substantially enhance the device specific capacity as well as energy density and power density.

According to the materials involved, paper‐like electrodes can be divided into four categories: graphene‐based paper‐like electrodes, CNT‐based paper‐like electrodes, carbon‐fiber paper‐like electrodes and carbon‐free paper‐like electrodes. Here we summarize and discuss the fabrication methods for each type of paper‐like electrode.

### 3.1 Graphene‐Based Paper‐Like Electrodes

Since the first report using micromechanical cleavage method to produce graphene sheet in 2004 by Geim and Novoselov, graphene and graphene‐based nanocomposites have received tremendous attention both for the sake of fundamental research as well as their great potential for applications in energy storage and conversion systems.^103^ Compared with other materials, graphene has unique 2D structure, high electronic mobility (15000 cm^2^ V^−1^ s^−1^),^103^, ^104^ exceptional electric (10^6^ S m^−1^)^105^ and thermal (5300 W m^−1^ K^−1^)^106^ conductivities, excellent optical transmittance (97.7% in visible region),^107^ good mechanical strength (tensile strength 130 GPa and stiffness 1.5 × 10^8^ psi)^108^ and ultrahigh surface area (≈2630 m^2^ g^−1^).^109^ Its extraordinarily high surface area enables its high electric double‐layer capacitance as high as 550 F g^−1^ if the entire surface area can be fully utilized.^102^

Several physical and chemical methods can be utilized to synthesize graphene including micromechanical cleavage, chemical vapor deposition, liquid phase exfoliation, reduction of graphene oxide (GO), thermal deposition of silicon carbide (SiC), and un‐zipping carbon nanotubes.^110^ To realize the commercial potential of graphene, it is essential to develop a reliable, low‐cost and facile process for large‐scale fabrication of high quality graphene materials. Solution processing offers a simple yet effective approach for the fabrication of graphene electrodes. To successfully using solution‐processing approach to fabricate graphene electrodes, there are three steps: first, preparation of a completely exfoliated GO solution; homogeneous dispersion of high‐quality graphene in a solvent; third, fabrication of graphene film. In 1958, Hummers et al. invented an effective oxidative method to produce GO solution, which is later called ‘Hummers method'.^111^ In 2008, Li et al. reported a facile method to make chemically converted graphene sheets to form stable aqueous dispersions.^112^ These studies pave a way for fabricating graphene and graphene‐based films. Graphene dispersions or graphene‐based hybrid materials suspension can be well processed into electrodes using various techniques, including dip coating,^113^ rod coating,^114^ spray coating,^115^ inkjet printing,^116^ spin coating,^117^ screen printing,^118^ gravure printing,^119^ blade coating,^120^ electrospinning,^121^ electrodeposition,^122^ vacuum filtration,^123^ drop casting,^124^ interfacial deposition,^124^ Langmiur‐Blodgett deposition^125^ and layer‐by‐layer assembly.^126^ Depends on the targeted properties and size of the graphene electrode, different approaches should be used.^102^

#### 3.1.1 Graphene Paper‐Like Electrodes

**Figure 5:**
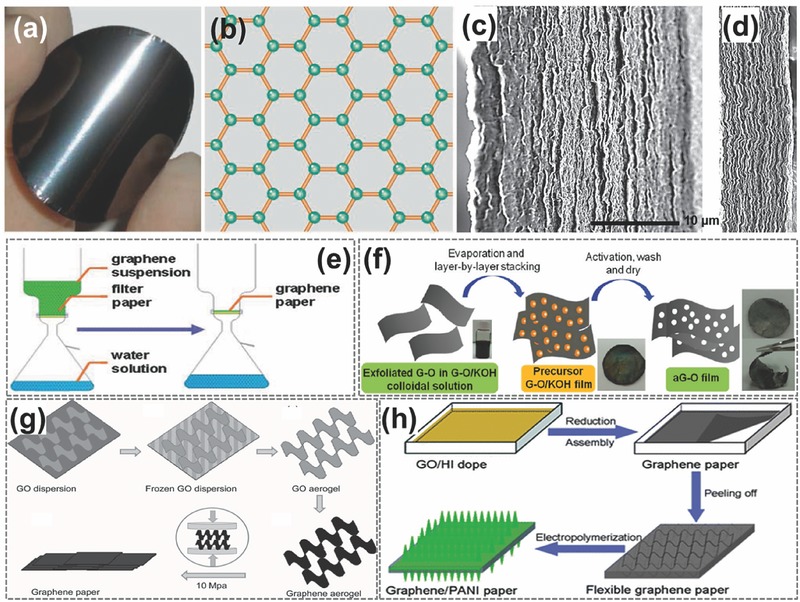
Schematic diagrams of four approaches for making graphene paper electrodes. a) Simple structure, great potential. Shiny and flexible graphene paper is formed by controlled restacking of graphene sheets. b) In graphene, carbon atoms (green dots) are bonded together through sp^2^ hybridization (orange lines). Reproduced with permission.^50^ Copyright 2008, American Association for the Advancement of Science. c,d) SEM images of cross sections of the obtained liquid electrolyte‐mediated chemically converted graphene films containing (c) 78.9 volume percent (vol. %) and (d) 27.2 vol. % of H_2_SO_4_, respectively, corresponding to ρ = 0.42 g cm^−3^ and ρ = 1.33 g cm^−3^. Reproduced with permission.^123^ Copyright 2013, American Association for the Advancement of Science. e) Graphene paper was produced via the vacuum filtration of graphene dispersion. Reproduced with permission.^132^ Copyright 2013, American Chemical Society. f) Schematic illustration showing the experimental steps of activated reduced graphene oxide film. Reproduced with permission.^133^ Copyright 2012, American Chemical Society. g) Illustration of the formation process of graphene paper. Step 1, GO aqueous dispersion. Step 2) GO dispersion frozen at −50 °C. Step 3) GO aerogel obtained by freeze drying. Step 4) Under vacuum. Step 5) Graphene aerogel obtained by treating Go aerogel at 200 °C in air. Step 6) Mechanical pressing of the graphene aerogel to form graphene paper. Reproduced with permission.^134^ h) Schematic illustrations of the formation process of graphene–PANI paper. Reproduced with permission.^135^ Copyright 2013, Royal Society of Chemistry.

Since the first graphite oxide paper reported in 2007 by Ruoff et al. using vacuum filtration method, free‐standing paper‐like graphene‐based materials have been extensively investigated.^127^ The high flexibility and ease of integration with other components make them ideal candidates for flexible energy storage devices (**Figure** **5**a,b).^50^

Free‐standing flexible graphene paper electrodes were first applied in Li‐ion battery and supercapacitor in 2009.^128^, ^129^ When employed as an electrode in Li‐ion battery, however, an enormous irreversible capacity was observed.^128^ The discharge capacity dropped drastically from 680 mAh g^−1^ in the first cycle to 84 mAh g^−1^ in the second cycle, only 12.4% of the initial capacity was retained. It was believed to be due to the irreversible reaction between the residual oxygen‐containing groups in the graphene paper and lithium. When graphene paper was employed as supercapacitor electrode, it displayed a gravimetrical capacitance of 147 F g^−1^ and volumetric capacitance of 64 F cm^−3^.^129^ Considering the small surface area of 94 m^2^ g^−1^ for the graphene paper, this high specific capacitance was attributed to the pseudocapacitance of oxygen functional groups (14%) on graphene surface. However, most of other graphene paper electrodes have capacitance typically smaller than 100 F g^−1^ because of severe restacking of graphene flakes and the low concentration of surface oxygen‐containing functional groups.^123^, ^130^, ^131^

Rational structural design of graphene paper electrode is critical for improving charge storage performance (Figure 5c–h). Graphene films with open pores allow fast ion diffusion and help to achieve both high capacitance and high rate capability. However, when graphene films are packed, the strong π‐π interaction between graphene sheets tend to restack to graphite‐like powders or films, which severely decrease the accessible surface area and ion diffusion rate, and thus, limiting both gravimetric capacitances and charge/discharge rates.

Constructing 3D graphene films with hierarchical pores is an effective way to enhance the energy storage performance. El‐Kady et al. fabricated a laser‐scribing graphene film with open porous structure, which can mitigate these challenges to increase the accessible surface area and boost the gravimetric capacitance up to 276 F g^−1^.^136^ Xu et al. reported a holey graphene framework film with a high packing density, open porous structure and efficient ion transport pathway to simultaneously with a high gravimetric capacitance (298 F g^−1^) and a high volumetric capacitance (212 F cm^−3^).^137^

Heteroatom doping in graphene can also improve the electrode performance. Doping graphene with oxygen (O), nitrogen (N), sulfur (S) and boron (B) have been proved to be effective in enhancing the performance of graphene paper for energy storage.^110^, ^138^, ^139^, ^140^, ^141^ Lu et al. successfully fabricated N‐doped graphene papers.^139^ Due to improved electrical conductivity and extra capacitance contributed from the pyridine‐like N dopants, the N‐doped graphene paper displayed a high specific capacitance of 280 F g^−1^, which is much higher than the un‐doped graphene paper with capacitance only 29 F g^−1^. Akhter et al. further demonstrated a N‐, S‐ codoped graphene paper electrode, which exhibited an excellent capacitive performance of 305 F g^−1^.^140^

Although graphene paper can achieve high specific capacitance over 200 F g^−1^ in supercapacitors, their low mass densities (0.05–0.75 g cm^−3^) make their volumetric capacitance rather modest (≈10–110 F cm^−3^).^123^ Yang et al. took the advantage of capillary compression of adaptive graphene gel films in the presence of a nonvolatile liquid electrolyte and enabled sub‐nanometer scale integration of graphene sheets with electrolytes to form highly packed graphene film electrode with a continuous ion transport network.^123^ This highly packed electrolyte‐mediated chemically converted graphene films (1.25–1.33 g cm^−3^) yielded a high volumetric capacitance of 255.5 F cm^−3^ in aqueous electrolyte and 261.3 F cm^−3^ in organic electrolyte.

#### 3.1.2 Graphene‐Based Composite Paper‐Like Electrodes

One of the most important approaches to manufacture high performance graphene‐based electrodes is to synthesize composite structures of graphene and pseudo‐capacitive materials such as conducting polymers and metal oxides. In this part, we will mainly focus on the different methods in fabricating various graphene‐based composite paper‐like electrodes.

##### 3.1.2.1 Vacuum Filtration:

Vacuum filtration is a high scalable, low cost and easy process that has been widely used for making the free‐standing paper‐like graphene‐based films. Vacuum filtration setup usually consists of Buchner funnel, filter membrane and vacuum system. Different kinds of filter membrane such as anodic aluminum oxide (AAO) filter membrane, PVDF membrane, PTFE membrane and cellulose membrane have been used in making graphene‐based papers.^112^, ^142^, ^143^, ^144^ Free‐standing graphene‐based paper can be produced by peeling off from the membrane after filtering and drying.

Graphene composite electrodes can be fabricated by first filtering a graphene paper and then deposit another pseudocapacitive material on graphene paper using other methods such as electrodeposition or chemical vapor deposition. Alternatively, the solution suspension of graphene and pseudocapacitive materials can be filtered simultaneously. These two approaches allow easy tuning of the film property by controlling the ratio between components as well as the total mass of active materials. The second approach is more popular recently, since it enables better mixing of graphene and pseudocapacitive materials.

**Figure 6:**
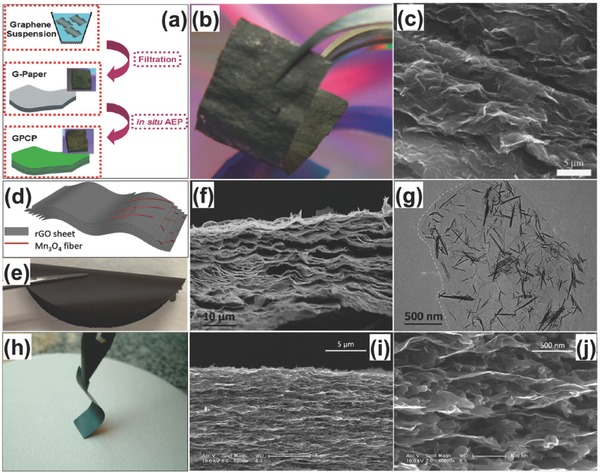
Flexible graphene‐based hybrid paper electrodes. a) Schematic diagram showing how MnO_x_ nanofibers are embedded among rGO sheets. b) Pictures of as filtered MnO_x_/GO nanohybrid paper rolled freely. c) SEM images of MnO_x_/GO nanohybrid paper: from cross‐sectional view. d) TEM images of MnOx nanofibers embedded in an rGO sheet, with part of the rGO sheet's edge marked out. Reproduced with permission.^145^ Copyright 2010, American Chemical Society. e) Digital photograph of a flexible graphene/polyaniline nanofiber composite film (G‐PNF). f,g) Cross‐section SEM images of G‐PNF. Reproduced with permission.^146^ Copyright 2010, American Chemical Society. h) Illustrative fabrication process toward graphene/polyaniline composite paper (GPCP). i) Digital camera images of a flexible G‐paper. j) SEM image of the GPCP. Reproduced with permission.^129^ Copyright 2009, American Chemical Society.

Cheng et al. fabricated the graphene/PANI composite paper by vacuum filtration of the graphene followed by anodic electropolymerization of PANI film. This paper displayed a favorable tensile strength of 12.6 MPa and a stable electrochemical capacitance of 233 F g^−1^ and 135 F cm^−3^ (**Figure** **6**a–c). Hu et al. demonstrated a highly flexible Mn_3_O_4_/reduced graphene oxide (rGO) nanohybrid paper with high conductivity via vacuum filtration of MnO_x_/GO mixture and electrochemical reduction process (Figure 6d–g). Asymmetric supercapacitor device based on Mn_3_O_4_/rGO paper as the cathode and rGO paper as the anode is assembled and showed a high volumetric capacitance of 54.6 F cm^−3^ and a high volumetric energy and power density (5.5 mWh cm^−3^ and 10.95 W cm^−3^).^145^ Wu et al. prepared the graphene/PANI nanofiber composite flexible films by vacuum filtration of the mixed dispersions of both the graphene and PANI nanofibers (Figure 6h–j). The composite film has a layered structure with PANI nanofibers sandwiched between graphene layers. It showed a high conductivity of 550 S cm^−1^, which is 10 times higher than the pure PANI nanofiber film. This composite electrode showed both increased capacitance and enhanced stability compared to bare graphene film and PANI film.^146^

##### 3.1.2.2 Laser Scribing:

Laser scribing is a simple and scalable all‐solid‐state approach that can convert GO into highly conductive graphene.^147^ The scribing laser simultaneously reduce and exfoliate GO sheets to yield open porous network of graphene.^136^ Besides, this method can also precisely control the shape of final products by designing different patterns, which can be widely used in making microscale energy storage devices.^148^, ^149^, ^150^

El‐Kady et al. used the commercially available LightScribe DVD to obtain high conductive graphene film.^136^ The graphene film showed an excellent conductivity of 1738 S m^−1^ and large and accessible specific surface area of 1520 m^2^ g^−1^. Besides, this 3D porous structure prevents the agglomeration of graphene sheets which has been the major barrier in realizing the full potential of graphene‐based supercapacitors. The flexible device can work over a wide voltage window of 3 V in organic electrolyte and offered a specific capacitance up to 265 F g^−1^. This working window could be even expanded to 4 V under ionic liquid electrolyte and exhibited as specific capacitance of 276 F g^−1^. The supercapacitor device delivered a high energy density up to 1.36 mWh cm^−3^ and power density of 20 W cm^−3^.

Gao et al. employed the non‐toxic direct laser writing to fabricate in‐plane supercapacitors with three different geometries.^148^ All of the three structures are consist of the rGO‐GO‐rGO configuration, where GO is served as a separator and rGO works as active electrode materials. The concentric circular pattern gives the highest capacitance density over the parallel column and hair brush structures. This in‐plane circular design showed an energy density of 0.43 mWh cm^−3^ and power density of 9.4 W cm^−3^ and kept 70 % of its initial capacitance after 10000 cycles.

##### 3.1.2.3 Mayer‐Rod Coating:

Mayer‐rod coating is a well‐known coating technique widely used in the coating industry for making thin films in a continuous and controlled manner. Mayer rod is a metal bar with a wire wrapped around that used to draw a solution over a substrate surface. The diameter of the wire wound around the bar determines the thickness of the graphene film. This technique can be used to produce large‐area graphene film with tunable thickness.^114^, ^135^, ^151^

Liu et al. used Mayer‐rod coating method to produce a free‐standing and highly flexible graphene‐PEDOT/PSS film with large size (30 cm × 7 cm) and high mass loading (8.49 mg cm^−2^).^151^ The flexible film show high areal capacitance of 448 mF cm^−2^. The assembled solid‐state supercapacitor device displayed stable performance over 10000 cycles both under flat and 180° bending states.

##### 3.1.2.4 Sacrificial template:

Sacrificial template method has been widely used in the colloid synthesis, which prepare materials on pre‐existing templates. Graphene can also be fabricated on various templates, such as metal mesh, calcium carbonate (CaCO_3_), silicon dioxide etc.^26^, ^152^, ^153^, ^154^, ^155^ After removing the template, free‐standing hierarchical graphene structure can be obtained. To make graphene‐based flexible films, sacrificial templates, such as nanoporous copper foils and calcium carbonate particles, have been used.^152^, ^153^

Qin et al. reported the synthesis of a continuous hierarchical nanoporous graphene film by CVD growth of hydrogenated graphite (HG) coating on nanoporous copper, rapid catalytic pyrolysis of HG using nanoporous copper as catalyst and followed by the removal of these catalysts.^153^ The as‐prepared flexible hierarchical graphene film owns a large surface area (1160 m^2^ g^−1^). The symmetric solid‐state device shows a high specific capacitance (38.2 F cm^−3^), a high energy density (2.65 mWh cm^−3^) and power density (20.8 W cm^−3^) normalized to volume of the entire device. Meng et al. employed CaCO_3_ as sacrificial template to fabricate hierarchical porous graphene film. PANI/graphene composite film was synthesized via polymerization of aniline monomer. This flexible electrodes achieved a high rate capability of 94% from 0.5 to10 A g^−1^.^152^

In addition to these methods and examples, there are other approaches for fabricating high‐performance graphene‐based flexible electrode for energy storage systems, such as layer‐by‐layer self‐assembly,^140^ pulse‐electropolymerization,^156^ and screen printing,^118^ which have been summarized in **Table** **2**.

**Table 2:** Graphene‐based paper‐like electrodes and their flexible energy storage devices

Electrodes	Synthesis methods	Device	Performance				Ref.
			Capacitance/Capacity	Power Density	Energy Density	Cycling Stability	
Graphene paper	Laser scribing	SC	276 F g^−1^	20 W cm^−3^	1.36 mWh cm^−3^	96.5% after 10000 cycles	136
Graphene paper	Frozen and pressing	SC	172 F g^−1^	–	–	99% after 5000 cycles	134
		LIB	864 mAh g^−1^	–	–	65.7% after 100 cycles	
Mesoporous graphene paper	Vacuum filtration	Li‐S*	1393 mAh g^−1^	–	–	49.5% after 50 cycles	157
Graphene hydrogel film	Hydrothermal reduction	SC	187 F g^−1^	0.37 kW kg^−1^	0.61 Wh kg^−1^	91.6% after 10000 cycles	158
	Pressing						
Porous graphene film	Freeze‐casting	SC	284.2 F g^−1^	282 kW kg^−1^	9.9 Wh kg^−1^	97.6% after 10000 cycles	159
Holey graphene film	Hydrothermal etching	SC	298 F g^−1^	7.46 kW kg^−1^	35.1 Wh kg^−1^	95% after 20000 cycles	137
			212 F cm^−3^	10.6 kW L^−1^	49.2 Wh L^−1^		
Nanoporous graphene film	Sacrificial template	SC	305 F g^−1^	20.8 W cm^−3^	2.65 mWh cm^−3^	94% after 10000 cycles	153
			95.4 F cm^−3^				
Porous activated rGO film	Vacuum filtration	SC	120 F g^−1^	500 kW kg^−1^	26 Wh kg^−1^	95% after 2000 cycles	133
rGO film	Laser writing	SC	0.51 mF cm^−2^	1.7 W cm^−3^	0.43 mWh cm^−3^	70% after 10000 cycles	148
rGO film	Photo reduction	SC	275 F g^−1^	–	–	98% after 100 cycles	160
N‐doped graphene paper	Vacuum filtration	SC	280 F g^−1^	–	–	99.4% after 40000 cycles	139
N/S‐codoped graphene paper	Layer‐by‐layer Self‐assembly	SC	305 F g^−1^	17.76 Wh kg^−1^	28.44 Wh kg^−1^	95.4% after 10000 cycles	140
			188 F cm^−3^				
PPy‐graphene film	Pulse‐electropolymerization	SC	237 F g^−1^	1.18 kW kg^−1^	33 Wh kg^−1^	–	156
PANI‐graphene paper	Vacuum filtration Electrodeposition	SC	233 F g^−1^	–	–	109% after 1500 cycles	129
			135 F cm^−3^				
PANI nanofiber‐graphene paper	Vacuum filtration	SC	210 F g^−1^	3.3 kW kg^−1^	18.8 Wh kg^−1^	79% after 800 cycles	146
PANI‐graphene paper	Vacuum filtration	SC	489 F g^−1^	–	–	96% after 500 cycles	161
	Chemical polymerization						
PANI‐graphene film	Screen printing	SC	269 F g^−1^	454 kW kg^−1^	9.3 Wh kg^−1^	203% after 1000 cycles	118
Graphene‐PANI‐graphene film	Vacuum filtration	SC	390 F g^−1^	2.4 kW kg^−1^	10.4 Wh kg^−1^	82% after 5000 cycles	162
PANI‐MnO_2_‐graphene	Vacuum filtration	SC	636.5 F g^−1^	–	–	85% after 10000 cycles	131
	Electrodeposition polymerization						
PANI‐3D‐graphene film	Sacrificial template polymerization	SC	385 F g^−1^	–	–	90% after 5000 cycles	152
PEDOT‐graphene film	Bar‐coating	SC	448 mF cm^−2^	3.59 kW kg^−1^	2.83 Wh kg^−1^	95% after 10000 cycles	151
			81.9 F g^−1^				
Si‐graphene paper	Vacuum filtration	LIB	3200 mAh g^−1^	–	–	83% after 150 cycles	163
Co_3_O_4_‐graphene paper	Vacuum filtration	LIB	1356 mAh g^−1^	–	–	61.9% after 40 cycles	164
Fe_2_O_3_‐graphene paper	Vacuum filtration	SC	178.3 F cm^−3^	6.21 W cm^−3^	56 mWh cm^−3^	83.1% after 10000 cycles	165
Fe_3_O_4_‐graphene paper	Vacuum filtration	LIB	1555 mAh g^−1^	–	–	85% after 50 cycles	166
MnO_2_‐graphene paper	Vacuum filtration annealing	SC	256 F g^−1^	–	–	74% after 1000 cycles	167
MnO_2_‐graphene paper	Vacuum filtration	SC	897 mF cm^−2^	3.8 mW cm^−2^	35.1 µWh cm^−2^	78% after 3600 cycles	168
Mn_3_O_4_‐graphene paper	Vacuum filtration	SC	54.6 F cm^−3^	10.95 W cm^−3^	5.5 mWh cm^−3^	95% after 6000 cycles	145
SnO_2_‐graphene paper	Vacuum filtration	LIB	526 mAh g^−1^	–	–	83.4% after 25 cycles	169
SnO_2_‐N‐graphene paper	Vacuum filtration	LIB	918 mAh g^−1^	–	–	63% after 50 cycles	170
MoO_3_‐graphene film	Vacuum filtration	LIB	291 mAh g^−1^	–	–	59.1% after 100 cycles	171
Mo_2_N‐graphene paper	Vacuum filtration NH_3_ annealing	SC	142 mF cm^−2^	0.035 W cm^−3^	1.05 mWh cm^−3^	85.7% after 4000 cycles	172
MoS_2_‐graphene paper	Vacuum filtration	SIB	347 mAh g^−1^	–	–	90% after 15 cycles	173
V_2_O_5_‐graphene paper	Hydrothermal reaction vacuum filtration	SC	21.3 F g^−1^	425 W kg^−1^	8.5 Wh kg^−1^	60% after 50 cycles	174
V_2_O_5_‐graphene paper	Vacuum filtration	SC	512 mF cm^−2^	625 W kg^−1^	13.3 Wh kg^−1^	90% after 8000 cycles	175
				4.17 mW cm^−2^	89 µWh cm^−2^		
V_2_O_5_·H_2_O‐graphene film	Vacuum filtration	SC	11.7 mF cm^−2^	10 µW cm^−2^	1.13 µWh cm^−2^	95% after 2000 cycles	176
VOPO_4_‐graphene film	Layer‐by‐layer	SC	8.36 mF cm^−2^	5.2 mW cm^−2^	1.7 mWh cm^−2^	96% after 2000 cycles	177
TiO_2_‐graphene paper	Vacuum filtration	LIB*	236 mAh g^−1^	–	–	99% after 90 cycles	178
Nb_2_O_5_‐graphene paper	Vacuum filtration	LIC*	160 mAh g^−1^	32 W kg^−1^	106 Wh kg^−1^	110.8% after 1000 cycles	179
T‐Nb_2_O_5_‐graphene paper	Hydrothermal reaction	SC	620.5 F g^−1^	18 W kg^−1^	47 Wh kg^−1^	94.8% after 1700 cycles	180
	Vacuum filtration		961.8 F cm^−3^				
CuO‐graphene paper	Vacuum filtration	LIB	782.3 mAh g^−1^	–	–	94.2% after 50 cycles	181
MnO_2_‐PPy‐graphene film	Electrodeposition	SC	600 F g^−1^	13 kW kg^−1^	28 Wh kg^−1^	92% after 5000 cycles	182
RuO_2_‐graphene film	Laser‐scribing	SC	1139 F g^−1^	81.4 kW kg^−1^	55.3 Wh kg^−1^	93% after 4000 cycles	147
			158 F cm^−3^				
Ni(OH)_2_‐grapheen paper	Vacuum filtration	SC	573 F g^−1^	17 kW kg^−1^	18 Wh kg^−1^	158% after 20000 cycles	183
			655 F cm^−3^				
β‐Ni(OH)_2_‐graphene film	Vacuum filtration	SC	660.8 F cm^−3^	–	–	100% after 2000 cycles	184
			3.3 mF cm^−2^				
Co(OH)_2_‐graphene film	Vacuum filtration	SC	20 F g^−1^	–	–	100% after 5000 cycles	185
		LIB	520 mAh g^−1^	–	–	23.1% after 200 cycles	
Li‐rGO film	Spark reaction	LIB	3390 mAh g^−1^	–	–	90% after 100 cycles	186
Carbon black‐graphene paper	Vacuum filtration	SC	112 F g^−1^	–	–	94% after 3000 cycles	187
3D CNT‐graphene film	CVD	SC	–	10.3 kW kg^−1^	22.8 Wh kg^−1^	90.2% after 10000 cycles	188

*Notes: Lithium‐ion capacitor (LIC), Lithium‐sulfur batteries (Li‐S), Sodium‐ion batteries (SIB).

### 3.2 CNT‐Based Paper‐Like Electrodes

Carbon nanotubes (CNTs) are well‐ordered hollow graphite nanomaterials consisted of cylinders of sp^2^‐hybridized carbon atoms. According to the number of layers of graphene sheets ‘rolled' into tubes, CNTs can be classed as sing‐walled carbon nanotubes (SWCNTs) and multi‐walled carbon nanotubes (MWCNTs). Diameters of SWCNTs and MWCNTs are typically 0.8 to 2 nm and 2 to 100 nm, respectively. The length of CNTs ranges from less than 100 nm to several centimeters.^189^

CNTs were first discovered by Iijima in 1991.^190^, ^191^ Since then, it has aroused the world‐wide research interest due to its high aspect ratio, high mechanical strength, large surface area, excellent chemical and thermal stability, and rich electronic and optical properties.^189^, ^192^, ^193^ With their unique physical properties, such as high electrical conductivity (10^4^–10^5^ S cm^−1^) and good chemical and mechanical stability, CNTs have proven to be promising for electrochemical energy storage.^11^, ^30^, ^49^, ^194^, ^195^, ^196^ Both SWCNTs and MWCNTs have been explored as energy storage electrode materials.

#### 3.2.1 CNTs Paper‐Like Electrodes

In 2004, Morris et al. demonstrated a free‐standing SWCNTs paper electrode and applied them into Li‐ion battery for the first time.^197^ The laboratory cell based on this film is able to display a specific energy exceeding 600 Wh kg^−1^ and power density over 3 kW kg^−1^. In 2005, a CNT ‘buckypaper' was fabricated via filtrating of double‐walled CNTs.^198^ This paper‐like sheet is flexible and mechanically stable. Thereafter, numerous reports on CNTs and CNTs‐based paper electrodes have been published.

Recently, a free‐standing flexible SWCNTs film was prepared by a floating chemical vapor deposition method.^199^ The specific capacitance of supercapacitor based on these films is about 35 F g^−1^. Its power density is 197.3 kW kg^−1^. Ultrathin MWCNT film was also fabricated via layer‐by‐layer assembly process.^200^ It achieved a high specific capacitance (159 F g^−1^) and volumetric capacitance (132 F cm^−3^).

**Figure 7:**
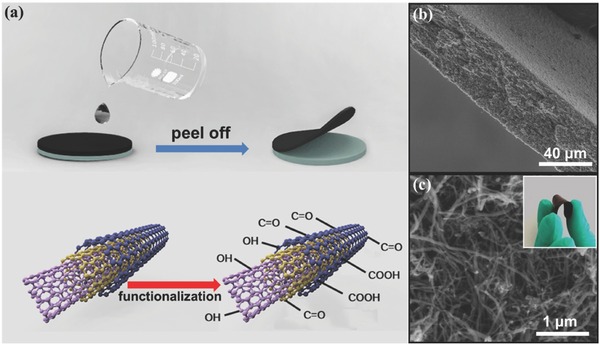
Freestanding functionalized carbon nanotubes (CNTs)‐based film. a) Schematic of the fabrication procedure for functionalized freestanding CNTs films. b) Cross‐section SEM image of the functionalized freestanding CNTs film. c) Enlarged cross‐section SEM image of the functionalized freestanding CNTs films. The inset is digital image of a functionalized freestanding CNTs film. Reproduced with permission.^49^ Copyright 2014, Elsevier.

Introducing pseudocapacitance into carbonaceous materials can greatly enhance their overall performance for charge storage. Xiao et al. used vacuum filtration method to fabricate flexible freestanding CNT films and then adopted an electrochemical method to add redox active functional groups onto CNT films (**Figure** **7**).^49^ Oxygen‐containing groups, especially carboxylate (–COOH) group played an important role in enhancing the supercapacitor performance. The functional CNT film showed an excellent areal capacitance of 150 mF cm^−2^, much larger than the bare CNT films with areal capacitance only about 40 mF cm^−2^. Likewise, the performance of CNT film electrodes can be enhanced by integrating other high electrochemically active materials.^33^, ^84^, ^96^, ^201^

#### 3.2.2 CNT‐Based Composite Paper‐Like Electrodes

One of the most widely adopted strategy to make high‐performance electrodes for energy storage systems is incorporating high conductive and stable carbon materials with high specific capacity/capacitance materials such as conducting polymers, metal oxides, metal nitrides and metal carbides. These composites take the advantages of both the high conductivity of carbon and high theoretical capacity/capacitance of those less conductive pseudocapacitive materials, which could achieve better performance than each individual component.

The unique high aspect ratio and fast electron migration along 1D direction of CNTs offer the paper‐based 3D CNT conductive network great potential in the applications for Li‐ion storage devices and supercapacitors. Various approaches can be used to fabricate CNT‐based free‐standing flexible paper‐like electrodes, such as vacuum filtration, doctor blade, chemical vapor deposition, and layer‐by‐layer assembly, etc.

##### 3.2.2.1 Vacuum Filtration:

Vacuum filtration is not only effective in producing graphene‐based paper‐like electrodes with 2D graphene nanosheets, but also useful in fabrication of paper‐like electrodes with 1D CNTs. By varying the components of the filtration suspension, different composite films can be obtained. The high aspect ratio property of CNTs guaranties the flexibility of the film. The 3D conductive network consists of these CNTs allows effective charge transport and function as a 3D current collector. Besides, this conducing network can also enclose the other active materials within this 3D conductive network and achieve a free‐standing flexible electrode without the need of insulated polymeric binders. By eliminating the conducting agents like carbon black and binders, these flexible free‐standing CNT‐based films are anticipate to have higher gravimetric and volumetric capacity/capacitance than conventional charge storage devices.

**Figure 8:**
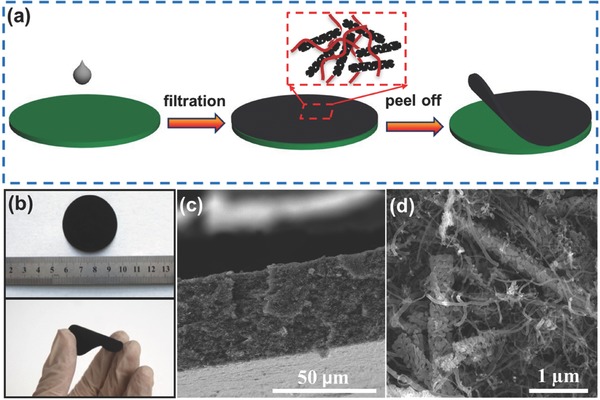
Freestanding mesoporous VN/CNT hybrid film. a) Schematic fabrication procedure of the paper‐like freestanding mesoporous VN nanowires/CNT (MVNN/CNT) hybrid electrodes. b) Digital images of paper‐like freestanding MVNN/CNT hybrid electrodes. c) Cross‐section SEM image of the freestanding MVNN/CNT hybrid electrode. d) Enlarged cross‐section SEM image of freestanding MVNN/CNT hybrid electrode. Reproduced with permission.^201^

Xiao et al. reported a free‐standing mesoporous VN/CNT hybrid electrode prepared by vacuum filtration of a solution mixture of VN nanobelts and CNTs (**Figure** **8**).^201^ Both VN and CNT have excellent electrical conductivity that ensures its application as electrode in solid‐state supercapacitor without the need of any current collector. This light‐weight device (15 mg including electrodes, separator and electrolyte) exhibited a high volume capacitance of 7.9 F cm^−3^ and energy density and power density of 0.54 mWh cm^−3^ and 0.4 W cm^−3^.

**Figure 9:**
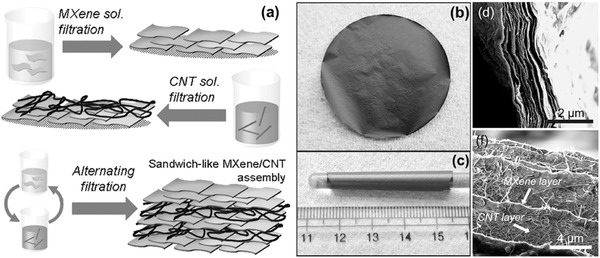
Flexible MXene/carbon nanotube composite paper. a) Schematic showing the preparation of the sandwich‐like MXene/CNT papers used herein. b,c) Digital photographs showing a flexible and free‐standing sandwich‐like MXene/CNT paper (b) and wrapping of latter around a 5‐mm‐diameter glass rod (c). d,e) Cross‐sectional SEM images of sandwich‐like Ti_3_C_2_T_x_/SWCNT (d) and Ti_3_C_2_T_x_/MWCNT papers (e). Reproduced with permission.^202^

The performance of the filtered free‐standing CNTs‐based paper‐like composite electrodes can also be engineered by designing the sequence of adding different components. Zhao et al. proposed a facile route for fabricating flexible and sandwiched MXene/CNT composite paper electrodes through alternating filtration of MXene and CNT dispersions.^202^ They found that the sandwiched electrode structure exhibited significantly enhanced electrochemical performance compared to pure MXene and randomly mixed MXene/CNT papers (**Figure** **9**).

##### 3.2.2.2 Doctor Blade:

Doctor blade, also known as knife coating, is a well‐established technique for fabricating large‐area films over rigid or flexible substrates. The method is widely used in the laboratories to make CNT and graphene films for batteries and supercapacitors. In a typical procedure, the active material slurry is transferred to substrate by moving the blade over a flat base. The variable thickness of the films is determined by the gap distance between the blade and substrate. Recently, this technique was used for the preparation of flexible free‐standing CNT and graphene‐based paper‐like films.^102^, ^147^, ^203^, ^204^

**Figure 10:**
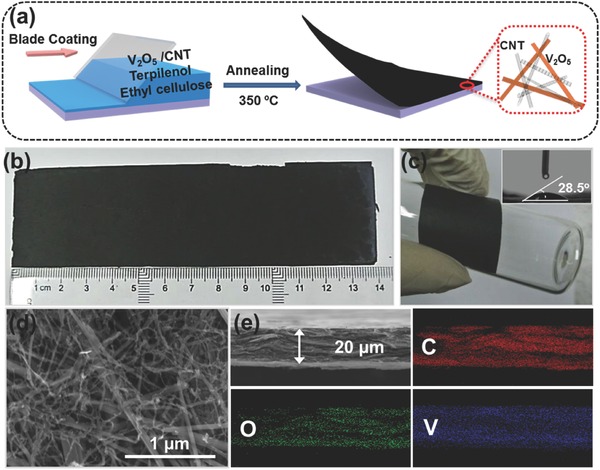
Scalable free‐standing V_2_O_5_/CNT film. a) Schematic diagram illustrating the processes for preparing V_2_O_5_/CNT free‐standing film; b) Optical image of V_2_O_5_/CNT film with length over 13 cm; c) Optical image of highly dense V_2_O_5_/CNT film cover the surface of a bottle, inset is the contact angle of this film; d) SEM image of highly dense V_2_O_5_/CNT film; e) EDX mapping images (carbon, oxygen, and vanadium mapping). Reproduced with permission.^33^

Wu et al. presented the fabrication of scalable V_2_O_5_/CNT freestanding films using a continuous doctor blade coating process (**Figure** **10**).^33^ The process is facile and cost‐effective while the obtained V_2_O_5_/CNT hybrid composite films have a high packing density of about 2.5 g cm^−3^ after pressing treatment. These films exhibited high volumetric capacitances around 460 F cm^−3^ and the symmetric capacitor based on these film electrodes showed a high volumetric energy density of 41 Wh L^−1^ within a wide voltage window of 1.6 V.

##### 3.2.2.3 Chemical Vapor Deposition:

Chemical vapor deposition is most commonly used approach for synthesizing CNTs, including SWCNTs and MWCNTs. Normally, CNTs grow on the substrate with metal catalyst nanoparticles and form 3D nanotube array structures.^192^ To produce paper‐like free‐standing CNT paper, these CNTs should be first dispersed into solution with surfactants, followed by vacuum filtration, doctor blade or other fabrication process. The presence of surfactants will considerably lower the electrical conductivity of the integrated CNT film. Floating catalyst chemical vapor deposition (FCCVD) is a facile method to directly produce CNT film. Compared with the traditional CVD methods which fix catalysts on substrates, the catalyst particles in FCCVD method are continuously formed in the reactor and catalyst deactivation problem is avoided. The catalysts are introduced into the hot reaction zone by three methods: 1) a syringe process using catalyst dissolved in carbon source, 2) sublimation of catalyst at elevated temperatures or 3) a gaseous catalyst source such as ferrocene.^205^ Recent research showed that the CNTs film produced via FCCVD displays high electrical conductivity and can be easily integrated with other materials to fabricate hybrid electrodes for flexible energy storage systems.^206^, ^207^

Niu et al. demonstrated a ‘skeleton/skin' SWCNTs/PANI structure by electrochemical polymerization directly on SWCNTs films prepared by FCCVD method. This unique continuous structure ensures these hybrid films have much higher conductivity and better electrochemical performance compared to SWCNTs/PANI composite films prepared by post‐deposition of PANI on SWCNTs films. The conductivity of these films can reach about 1138 S cm^−1^. Supercapacitors fabricated using these films can achieve both high energy density (131 Wh kg^−1^) and power density (62.5 kW kg^−1^).^207^ A recent research further showed that the isotropically buckled CNT film synthesized via FCCVD method can be applied to omnidirectionally stretchable supercapacitors and displays high performance.^206^

Except for the electrode materials and structures mentioned above, lots of CNT‐based and CNT‐composite‐based electrodes have been prepared via various facile methods. A detailed summary of CNT‐based paper‐like electrodes and their flexible energy storage devices is depicted in **Table** **3**.

**Table 3:** CNT‐based paper‐like electrodes and their flexible energy storage devices

Electrodes	Synthesis methods	Device	Performance				Ref.
			Capacitance/Capacity	Power Density	Energy Density	Cycling Stability	
SWCNT film	FCCVD*	SC	35 F g^−1^	197.3 kW kg^−1^	43.7 Wh kg^−1^	–	199
MWCNT film	Layer‐by‐layer assembly	SC	159 F g^−1^	–	–	–	200
			132 F cm^−3^				
Functionalized CNT film	Vacuum filtration	SC	150 mF cm^−2^	4.2 W cm^−3^	1.5 mWh cm^−3^	90% after 10000 cycles	49
PPy‐CNT film	CVD	SC	494 F g^−1^	–	–	65.6% after 10000 cycles	208
	Electrodeposition		59.3 mF cm^−2^				
PPy‐CNT film	Vacuum filtration Electrodeposition	SC	0.28 F cm^−2^	–	–	95% after 10000 cycles	209
PPy‐MnO_2_‐CNT film	CVD	SC	351 F g^−1^	10 kW kg^−1^	39.7 Wh kg^−1^	94.4% after 10000 cycles	210
	In situ polymerization						
PANI‐CNT paper	Vacuum filtration Electrodeposition	SC	350 F g^−1^	2.19 kW kg^−1^	7.1 Wh kg^−1^	91.9% after 1000 cycles	211
PANI‐SWCNT film	Electrodeposition	SC	236 F g^−1^	62.5 kW kg^−1^	131 Wh kg^−1^	85% after 1000 cycles	207
PEDOT‐SWCNT film	Vacuum filtration	SC	133 F g^−1^	825 W kg^−1^	7 Wh kg^−1^	90% after 1000 cycles	212
Si‐PEDOT‐CNT film	Vacuum filtration	LIB	2180 mAh g^−1^	–	–	90% after 100 cycles	213
Si‐SWCNT paper	Vacuum filtration	LIB	3000 mAh g^−1^	–	–	60% after 50 cycles	214
	PLD*						
AC‐CNT paper	Vacuum filtration	SC	267.6 F g^−1^	7.3 kW kg^−1^	22.5 Wh kg^−1^	97.5% after 5000 cycles	215
MnO_2_‐CNT film	Water‐assisted CVD	SC	540 F g^−1^	1.5 kW kg^−1^	20 Wh kg^−1^	–	216
	Vacuum filtration						
Ti_3_C_2_‐CNT paper	Vacuum filtration	SC	390 F cm^−3^	–	–	114.1% after 10000 cycles	202
Ti_3_C_2_‐CNT paper	Vacuum filtration	SIB	421 mAh g^−1^	–	–	109% after 500 cycles	217
Porous Ti_3_C_2_‐CNT paper	Vacuum filtration	LIB	1250 mAh g^−1^	–	–	63.3% after 100 cycles	218
Nb_2_C‐CNT paper	Vacuum filtration	LIB	780 mAh g^−1^	–	–	116% after 100 cycles	219
MoO_3–x_/CNT paper	Vacuum filtration	SC	420 F g^−1^	–	–	90% after 5000 cycles	86
			420 F cm^−3^				
H_x_MoO_3–y_/CNT paper	Vacuum filtration	SC	350 F cm^−3^	6.8 W cm^−3^	1.1 mWh cm^−3^	93.3% after 4000 cycles	84
K_y_MoO_3–x_/CNT paper	Vacuum filtration	SC	374 F cm^−3^	6.5 W cm^−3^	0.91 mWh cm^−3^	89% after 10000 cycles	220
V‐MnO_2_‐CNT‐paper	Vacuum filtration	SC	439 F g^−1^	1.6 W cm^−3^	4.98 mWh cm^−3^	92% after 10000 cycles	88
V_2_O_5_‐CNT film	Doctor‐blade	SC	460 F cm^−3^	25 kW L^−1^	41 Wh L^−1^	79.8% after 5000 cycles	33
V_2_O_5_‐CNT film	Vacuum filtration	SIC*	35 F g^−1^	45 kW kg^−1^	38 Wh kg^−1^	80% after 900 cycles	221
V_2_O_5_‐CNT film	Hydrothermal reaction vacuum filtration	LIB	340 mAh g^−1^	–	–	76.5% after 50 cycles	222
VN‐CNT film	Vacuum filtration	SC	7.9 F cm^−3^	0.4 W cm^−3^	0.54 mWh cm^−3^	82% after 10000 cycles	201
In_2_O_3_‐CNT film	Vacuum filtration	SC	64 F g^−1^	7.48 kW kg^−1^	1.29 Wh kg^−1^	82.8% after 500 cycles	223
rNb_2_O_5_‐rGO‐CNT film	Vacuum filtration	SC	726.2 C g^−1^	–	–	87% after 3000 cycles	224
WO_3_‐CNT film	Vacuum filtration Evaporation	SC	2.6 F cm^−3^	30.6 mW cm^−3^	0.59 mWh cm^−3^	75.8% after 50000 cycles	225
Ni(OH)_2_‐CNT paper	Vacuum filtration Chemical bath deposition	SC	1144 F g^−1^	–	–	98% after 1000 cycles	203
PMTA/SWCNT‐SWCNT film	Filtration	LIB	163 mAh g^−1^	–	–	86.6% after 200 cycles	204
	Rolling						
Graphene‐PPy‐CNT film	Vacuum filtration	SC	211 F g^−1^	–	–	95% after 5000 cycles	226
			122 F cm^−3^				

*Notes: Floating catalyst chemical vapor deposition (FCCVD), Pulsed laser deposition (PLD), Sodium‐ion capacitor (SIC).

### 3.3 Carbon‐Fiber Paper‐Like Electrodes

Carbon‐fiber network consisted of interconnected carbon fibers with diameters ranging from tens of nanometers to several micrometers is another important kind of carbon‐based paper‐like electrodes. These carbon fiber network has high specific surface area, high electrical conductivity and highly scalable.^227^ Several approaches can be used to produce these 3D cross‐linked carbon structures, such as electrospinning, carbide derivation, CVD, etc.^28^, ^228^, ^229^, ^230^, ^231^, ^232^, ^233^, ^234^ Among these methods, electrospinning seems to provide the simplest approach in making nanofibers and microfibers with both solid and hollow interiors that are exceptionally long in length, uniform in diameter and diversified in composition.^227^ Normally, the electronspun carbon fiber systems are rigid and non‐flexible. However, by tuning synthetic conditions, flexible carbon‐fiber based paper‐like films can be obtained.

Mechanically flexible films consisting of electronspun carbon fibers were prepared by first electrospinning aqueous mixtures of natural alkali lignin with polyvinyl alcohol (PVA) followed by stabilization in air and carbonization in inert atmosphere.^234^ This flexible electrode yielded specific capacitance of 64 F g^−1^ and retained 90% of its initial capacitance after 6000 cycles.

**Figure 11:**
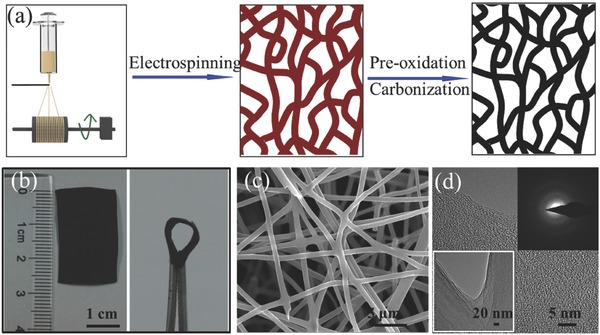
Flexible and cross‐linked N‐doped carbon nanofiber network film. a) Schematic of the preparation of cross‐linked carbon nanofibers. b) Photo image of the CLCF before and under folding; c) SEM and d) HRTEM images of the CLCF. Reproduced with permission.^228^ Copyright 2015, Elsevier.

Cross linking of electrospun carbon fibers can help to further increase its electrical conductivity.^235^, ^236^ Heteroatom doping can also contribute to the increased conductivity and the added pseudocapacitance for electric double‐layer capacitance carbon.^237^ Cheng et al. successfully fabricated flexible, porous, N‐doped carbon nanofibers network with strong cross‐linked structure between fibers via carbonization of electrospun polyacrylonitrile (PAN)/polyvinylpyrrolidone (PVP)/terephthalic acid (TPA) hybrid composite fibers (**Figure** **11**).^228^ Compared to non‐cross‐linked carbon nanofiber network, the cross‐linked N‐doped carbon nanofiber network owns a reduced resistance and enhanced capacitance (223.8 F g^−1^). In a more recent study, Cheng et al. reported that TPA can be used as pore forming agent in fabrication of flexible, porous carbon nanofibers network.^229^ This porous carbon fiber network exhibited increased specific capacitance (257.6 F g^−1^) and rate capability (64.4% from 0.5 A g^−1^ to 700 A g^−1^) compared to non‐porous carbon fiber network.

Carbide derivation is also an effective approach to develop carbon nanofiber network.^231^, ^232^ Presser et al. fabricated carbide‐derived carbon (CDC) via electrospinning of titanium carbide (TiC) and the following chlorination to remove Ti at elevated temperatures.^231^ The as‐prepared TiC‐CDC films show an average pore size of ≈1 nm and high specific surface area of 1390 m^2^ g^−1^. Besides, these mechanically flexible films can display a high gravimetric capacitance of 110 F g^−1^ in aqueous electrolyte and 65 F g^−1^ in organic electrolyte when directly used as electrode material for supercapacitor application.

Carbon‐fiber network films are good candidates to deposit other high capacity/capacitance active materials due to its open porous structure, high electrical conductivity and high mechanical stability. Yan et al. used electrospun carbon nanofiber (CNF) paper as substrate to polymerize polyaniline on its surface and successfully fabricated free‐standing flexible CNF/PANI composite paper.^238^ This paper electrode demonstrated a high specific capacitance of 638 F g^−1^ for CNF/PANI composite paper electrode, two times higher than the bare CNF paper electrode (317 F g^−1^). Miao et al. obtained a flexible porous carbon nanofiber (PCNF)/MoS_2_ core‐shell fiber film through a combination of electrospinning of carbon fiber networks and solvothermal coating of MoS_2_ nanosheet arrays.^239^ When the highly flexible PCNF/MoS_2_ composite film is used as a binder‐free anode in lithium‐ion battery, it exhibits a high specific capacity of 954 mAh g^−1^ and good cycling stability with almost 100% retention after 50 cycles.

The detailed compositions, synthesis methods of carbon fiber‐based paper‐like electrodes and their performances in flexible energy storage devices are summarized in **Table** **4**.

**Table 4:** Carbon fiber‐based paper‐like electrodes and their flexible energy storage devices

Electrodes	Synthesis methods	Device	Performance				Ref.
			Capacitance/Capacity	Power Density	Energy Density	Cycling Stability	
N‐carbon fiber paper	Electrospinning	SC	175 F g^−1^	1.2 kW kg^−1^	5.9 Wh kg^−1^	106% after 20000 cycles	228
Porous carbon nanofiber film	Electrospinning	SC	104.5 F g^−1^	600 W kg^−1^	3.22 Wh kg^−1^	94% after 2000 cycles	240
Porous cross‐linked carbon fiber paper	Electrospinning	SC	257.6 F g^−1^	56 kW kg^−1^	10.2 Wh kg^−1^	95% after 20000 cycles	229
Porous carbon nanofiber‐ ultrathin graphite film	CVD	SC	120 F g^−1^	23 W cm^−3^	2.4 mWh cm^−3^	96% after 5000 cycles	28
Carbon fiber‐graphene paper	Electrospinning Vacuum filtration	SC	197 F g^−1^	–	–	83.8% after 1500 cycles	241
Carbon nanofiber‐graphene paper	Electrospinning	SC	183 F g^−1^	–	–	92% after 4500 cycles	242
PANI‐carbon fiber paper	Electrospinning Polymerization	SC	638 F g^−1^	–	–	90% after 1000 cycles	238
Olivine‐carbon nanofiber	Electrospinning	LIB	152 mAh g^−1^	–	–	98.2% after 100 cycles	243
V_2_O_5_‐carbon fiber paper	Electrospinning Electrodeposition	SC	214 F g^−1^	156 W kg^−1^	45 Wh kg^−1^	80% after 1000 cycles	244
MoS_2_‐carbon fiber membrane	Electrospinning	LIB	954 mAh g^−1^	–	–	100% after 50 cycles	239
NiS‐carbon nanofiber membrane	Electrospinning	LIB	1149 mAh g^−1^	–	–	84.3% after 100 cycles	245

### 3.4 Carbon‐Free Paper‐Like Electrodes

Carbon allotropes including amorphous carbon,^246^ graphite,^76^ fullerene,^247^, ^248^ carbon nanotubes,^49^ and graphene^123^, ^249^ have been widely studied as electrode materials for energy storage. Different structures such as carbon film,^76^, ^250^ carbon foam,^154^, ^251^ carbon aerogel,^26^, ^252^ carbon hydrogel,^253^, ^254^ carbon paper,^255^, ^256^ and carbon cloth,^80^, ^91^ have been developed. Various morphologies like nanodots,^257^ nanotubes,^49^ nanoribbon,^258^ nanofiber,^228^ nanosheets,^109^ have also been explored to improve their performances. However, the theoretical capacitance/capacity of carbon materials is relatively low. For example, the theoretical capacitance of graphene for supercapacitor is 550 F g^−1^ and the theoretical capacity of graphite for Li‐ion battery is 372 mAh g^–1^.^102^, ^259^ These values are considerably lower than the theoretical values of most pseudocapacitive materials (>1000 F g^−1^).^13^, ^70^, ^87^ Thus, to develop free‐standing carbon‐free paper‐like electrodes for energy storage is attractive. Several methods have been reported in making such kind of electrodes, including vacuum filtration, rolling, electrospinning, anodization, electrodeposition, and sacrificial template methods, etc.

**Figure 12:**
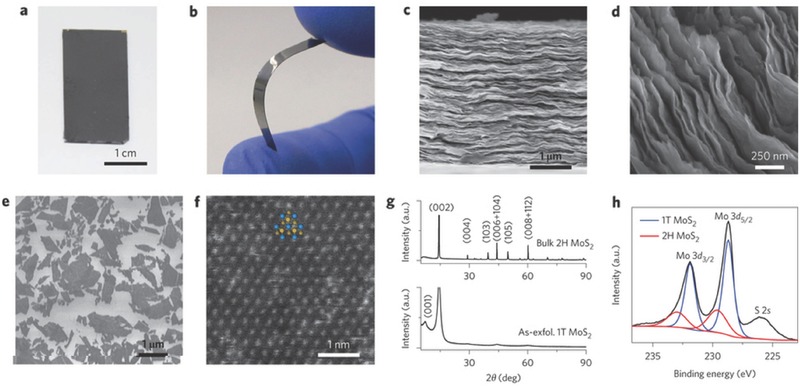
Chemically exfoliated 1T MoS_2_ electrodes. a,b) Photographs of electrodes consisting of a thick film of chemically exfoliated 1T MoS_2_ prepared by vacuum filtration and transferred onto rigid glass (a) and a flexible polyimide substrate (b). c) Side view of the electrode observed by scanning electron microscopy (SEM) showing the layered nature of the film made by restacking exfoliated MoS_2_ nanosheets. d) High‐magnification image of restacked MoS_2_ nanosheets. e) SEM image of as‐exfoliated monolayer 1T phase MoS_2_ nanosheets. f) High‐angle annular dark‐field scanning transmission electron microscope image of monolayer 1T phase MoS_2_. Inset: Atomic structure of 1T phase MoS_2_ (Mo and S atoms are displayed in blue and yellow, respectively). g) XRD of bulk MoS_2_ compared with as‐exfoliated restacked MoS_2_. h) High‐resolution X‐ray photoelectron spectrum from the Mo 3d region of as‐exfoliated 1T MoS_2_ (black). Contributions from 1T and 2H phase components in the Mo 3d spectrum are indicated by blue and red curves, respectively. Reproduced with permission.^260^ Copyright 2015, Nature Publishing Group.

High conductive nanomaterials could be used to fabricate high‐performance flexible free‐standing paper without the need of carbon materials as mechanical support or current collector. Acerce et al. successfully synthesized 1T phase MoS_2_ nanosheets and used vacuum filtration method to get a free‐standing pure MoS_2_ paper.^260^ These paper‐like electrodes achieved high volumetric capacitances ranging from 400 to 700 F cm^−3^ in a variety of aqueous electrolytes. These MoS_2_ paper electrodes achieved an even higher energy density (0.11 W cm^−3^) and power density (51 W cm^−3^) in organic electrolyte with operating working window up to 3.5 V (**Figure** **12**).

**Figure 13:**
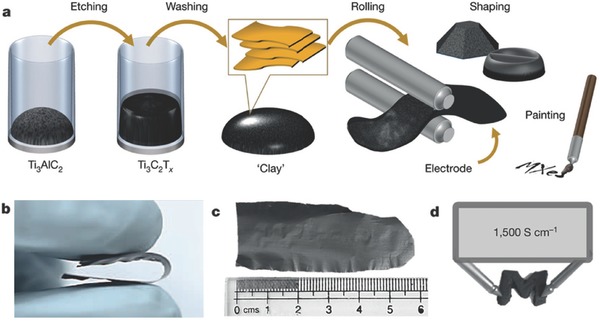
Free‐standing titanium carbide ‘clay' film. a) Schematic of MXene clay synthesis and electrode preparation. MAX phase is etched in a solution of acid and fluoride salt (step 1), then washed with water to remove reaction products and raise the pH towards neutral (step 2). The resulting sediment behaves like a clay; it can be rolled to produce flexible, freestanding films (step 3), moulded and dried to yield conducting objects of desired shape (step 4), or diluted and painted onto a substrate to yield a conductive coating (step 5). b) When dried samples (left, showing cross‐section and top view) are hydrated (right) they swell; upon drying, they shrink. c) Image of a rolled film. d) ‘Clay' shaped into the letter M (≈1 cm) and dried, yielding a conductive solid (labelled with the experimental conductivity of ‘clay' rolled to 5 mm thickness). The etched material is referred to as Ti_3_C_2_T_x_, where the T denotes surface terminations, such as OH, O and F. Reproduced with permission.^51^ Copyright 2014, Nature Publishing Group.

MXenes (of the fomula M_n+1_X_n_T_x_, where M is a transition metal, X is C and/or N, and T_x_ denotes surface functionalization) are a relatively young family of two‐dimensional (2D) materials.^51^, ^83^ MXenes are of high conductivity and can be fabricated into free‐standing films, which can be directly used as electrodes in Li‐ion batteries and supercapacitors. Ghidiu et al. synthesized highly conductive two‐dimensional titanium carbide ‘clay' and rolled them into free‐standing flexible Ti_3_C_2_T_x_ films (**Figure** **13**).^51^ The thickness of these films can be tuned. Their densities are in the range of 2.2–3.8 g cm^−3^. By employing these films as electrodes in supercapacitor, they showed a high volumetric capacitance over 900 F cm^−3^ and gravimetrical capacitance of 245 F g^−1^. The electrodes also demonstrated no measurable capacitance losses even after 10000 cycles.

**Figure 14:**
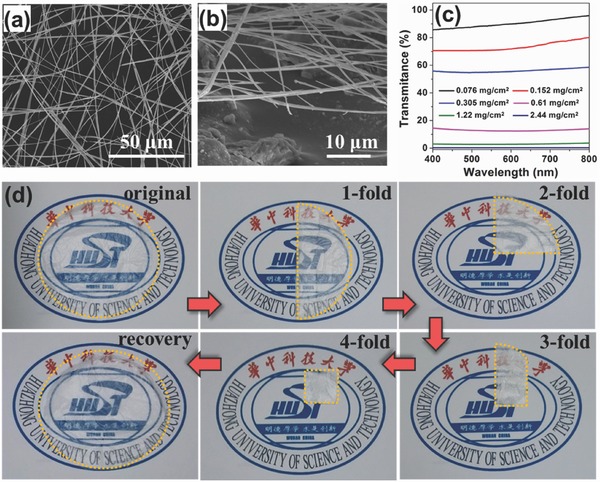
Ultrathin transparent MoO_3_ nanobelts nanopaper. a) SEM image of ultrathin transparent MoO_3_ nanobelts nanopaper. b) Side view of transparent MoO_3_ nanopaper. c) Transmittance spectra of MoO_3_ nanopapers with different mass loading. d) Optical images of transparent MoO_3_ nanopaper under different times of folding: Original, one folding, two folding, three folding, four folding, and full recovery. Reproduced with permission.^13^

In addition to these highly conductive materials, materials that are less conductive can also be employed as free‐standing paper electrode. Yao et al. reported an ultrathin, transparent, molybdenum trioxide (MoO_3_) nanopaper electrode via vacuum filtration.^13^ The ultrathin film with only two or three nanobelts in thickness allows not only efficient ion diffusion but also effective electron transport even MoO_3_ is not very conductive (**Figure** **14**). This nanopaper electrode delivers an excellent specific capacitance of 1198 F g^−1^ and long‐term cycling stability over 20000 cycles.

**Figure 15:**
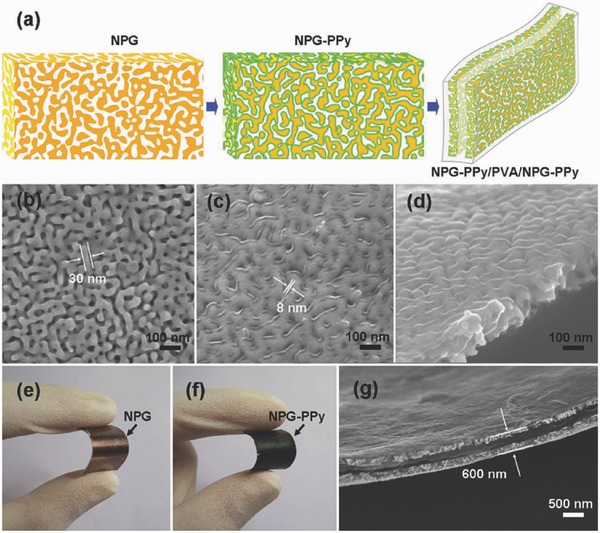
Flexible conducting polymer‐nanoporous metal film electrode. a) Schematic illustration of the fabrication process of the ultrathin flexible solid state supercapacitor. b,c) SEM images of nanoporous gold (NPG) and NPG‐PPy_8s_. d) SEM side view of NPG‐PPy_8s_. e,f) Digital pictures of macroscopic NPG and NPG‐PPy thin membranes. g) SEM cross sectional image of the as‐designed symmetric supercapacitor device. Reproduced with permission.^263^

Nanoporous metal paper is emerging as a new class of material for energy storage.^261^ They can be used as a highly conductive substrate for deposition of metal oxide or conducting polymers (**Figure** **15**).^262^, ^263^ Alternatively, they can also be transformed into metal oxide, metal nitride, metal carbide or metal fluoride and are directly employed as electrodes for flexible energy storage systems.^264^ The multifunctional properties of nanoporous metal papers make them particularly attractive for carbon‐free electrodes.

The research on carbon‐free paper‐like electrodes is just in its infancy, further research on both material selection and electrode architecture is essential. More carbon‐free paper‐like electrodes and their applications in flexible energy storage devices are summarized in **Table** **5**.

**Table 5:** Carbon‐free paper‐like electrodes and their flexible energy storage devices

Electrodes	Synthesis methods	Device	Performance				Ref.
			Capacitance/Capacity	Power Density	Energy Density	Cycling Stability	
Si nanowire paper	RFCVD*	LIB	2699 mAh g^−1^	–	–	38.4% after 100 cycles	265
MoO_3_ nanopaper	Vacuum filtration	SC	1198 F g^−1^	10.1 kW kg^−1^	22.89 Wh kg^−1^	96.5% after 20000 cycles	13
h‐MoO_3_ film	Vacuum filtration	SC	996 C g^−1^	21.3 W cm^−3^	0.3 Wh cm^−3^	92% after 6000 cycles	266
			1100 C cm^−3^				
MoO_3–x_ nanopaper	Vacuum filtration	SC	652 F cm^−3^	1.45 kW kg^−1^	8.3 Wh kg^−1^	85.7% after 25000 cycles	267
MoS_2_ nanopaper	Vacuum filtration	SC	650 F cm^−3^	51 W cm^−3^	0.11 Wh cm^−3^	93% after 10000 cycles	260
α‐MnO_2_ paper	Vacuum filtration	SC	118 F g^−1^	–	–	95.3% after 1000 cycles	268
TiO_2_ paper	Vacuum filtration	LIB	339 mAh g^−1^	–	–	100% after 400 cycles	269
TiO_x_N_y_ paper	Vacuum filtration	SC	120.9 F g^−1^	–	–	88% after 300 cycles	270
Ti_3_C_2_ paper	Vacuum filtration	SC	900 F cm^−3^	–	–	100% after 10000 cycles	51
			245 F g^−1^				
Ti_3_C_2_/PVA paper	Vacuum filtration	SC	528 F cm^−3^	–	–	85.9% after 10000 cycles	271
VN@C film	Vacuum filtration	SC	282 mF cm^−2^	2.72 W cm^−3^	0.97 mWh cm^−3^	91.8% after 12000 cycles	272
NiO‐Ni nanofiber paper	Electrospinning	LIB	1054 mAh g^−1^	–	–	92.8% after 1500 cycles	273
NiF_2_ film	Anodization	SC	66 mF cm^−2^	112 kW kg^−1^	384 Wh kg^−1^	150% after 10000 cycles	264
			733 F cm^−3^				
			358 F g^−1^				
PPy film	Sacrificial template	SC	316.2 F g^−1^	–	–	30.9% after 500 cycles	274
PEDOT film	Vacuum filtration	SC	120 F g^−1^	16.2 W cm^−3^	6.8 mWh cm^−3^	80% after 8000 cycles	275
NiMn nanoporous metal paper	Dealloying	SC	4.4 F cm^−2^	–	–	85% after 2000 cycles	261
			887 F cm^−3^				
MnO_2_‐nanoporous gold paper	Electrodeposition	SC	1145 F g^−1^	16 kW kg^−1^	57 Wh kg^−1^	85% after 1000 cycles	262
MnO_2_‐nanoporous Au‐MnO_2_ paper	Electrodeposition	SC	916 F g^−1^	–	–	97.1% after 3000 cycles	276

Note: *RFCVD: Radio frequency chemical vapor deposition.

## 4 Applications

The heavy demand of flexible electronics push the development of various flexible energy storage devices. Different types of energy storage devices have different scopes of application, depending on their electrochemical properties and working mechanism. For example, supercapacitors are suitable for electronics that required fast charging and discharging and long cycling stability, while lithium‐oxygen batteries fit the applications that require continuously and high energy supply. In this section, four different kinds of flexible paper‐based energy storages devices, including supercapacitors, Li‐ion batteries, Li‐S batteries and Li‐O_2_ batteries, will be introduced and discussed.

### 4.1 Flexible Supercapacitors

Supercapacitors, also known as ultracapacitors or electrochemical capacitors, have gained much research interest during the past decade. However, the concept of capacitor is not new. In 1879, Helmholtz first discovered the phenomenon of double‐layer capacitance.^277^ The first patent on double‐layer capacitor was filed by Becker in 1957.^278^ The first generation of commercial capacitors were manufactured by Panasonic/Matsushita in 1978 and NEC in 1980.^279^ Since then, enormous efforts have been devoted to the research on this ultrafast charging and discharging devices.

According to the different working mechanism, supercapacitors can be classified into electric double layer capacitors (EDLC) and pseudocapacitors.^280^ EDLCs store energy mainly by ion absorption at the interface between electrodes and electrolyte. Pseudocapacitors rely on the fast redox reactions at the electrode surface to collect and release charges. Carbonaceous materials are widely explored for EDLCs, while the conducting polymers and metal oxides are mainly used for pseudocapacitors. Because of their different charge‐storage mechanisms, EDLCs typically have excellent cycling stability (>200, 000 cycles) and power density, but relatively low in specific capacitance and energy density. Pseudocapacitors, on the other hand, are great in terms of energy density, but inferior in stability and power density. Due to chemical reactions are involved in pseudocapacitors, irreversible components will accumulate during cycling, leading to device deterioration.^20^ Recently, a new concept of Li‐ion capacitor by integrating EDLC electrode as cathode and pseudocapacitors electrode as anode has been proposed. It is anticipated that this kind of capacitors can achieve higher capacitance than EDLCs and better cycling stability than pseudocapacitors. Thus the energy density is improved without sacrificing the power density.^281^

Supercapacitors can be assembled as sandwich‐like devices and planar interdigital devices. On the basis of different types of electrolyte used, supercapacitors can also be classified into aqueous supercapacitors and solid‐state supercapacitors. As for the flexible solid‐state supercapacitors, gel‐based electrolytes are widely adopted, including PVA/H_3_PO_4_, PVA/H_2_SO_4_, and PVA/KOH, PVA/LiCl, etc.^12^, ^14^, ^80^, ^282^

**Figure 16:**
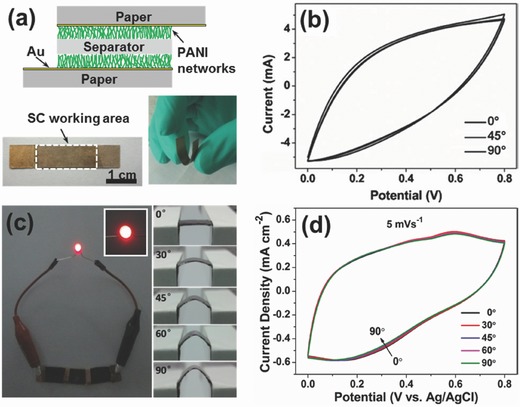
Flexible paper‐based solid‐state supercapacitors. a) The fabricated solid‐state SCs (upper) and photographs of the SC (down). b) CV scans of the all‐solid‐state SC at different curvature. Reproduced with permission.^32^ c) The left part is the optical picture of three solid‐state supercapacitors with 120 minutes PANI depositing graphite/polyaniline‐paper (G/PANI‐Paper) electrodes in series to light a red LED. The inset is the enlarged optical picture of the alight LED. The right part is optical pictures of the flexible solid‐state supercapacitors with 120 minutes PANI depositing G/PANI‐Paper electrodes at five bending states with bending angle of 0°, 30°, 45°, 60° and 90°, respectively. b) Cyclic voltammetry of the supercapacitors at the five bending states. Reproduced with permission.^14^ Copyright 2013, Elsevier.

For paper‐based and paper‐like electrodes, most of them are assembled into sandwich‐like supercapacitor configuration. Yuan et al. assembled the solid‐state supercapacitors by sandwiching a PVA/H_3_PO_4_ film between two identical PANI‐Au paper electrodes (**Figure** **16**a,b).^32^ PVA/H_3_PO_4_ film worked as both the electrolyte and the separator. This devices displayed good stability under different bending state. Likewise, Yao et al. fabricated the solid‐state supercapacitors via sandwiching a PVA/H_2_SO_4_ gel film between two symmetric PANI/graphite paper electrodes (Figure 16c,d).^14^ The cyclic voltammetry curves of the as‐fabricated devices almost did not change under different bending states, showing its excellent flexibility.

**Figure 17:**
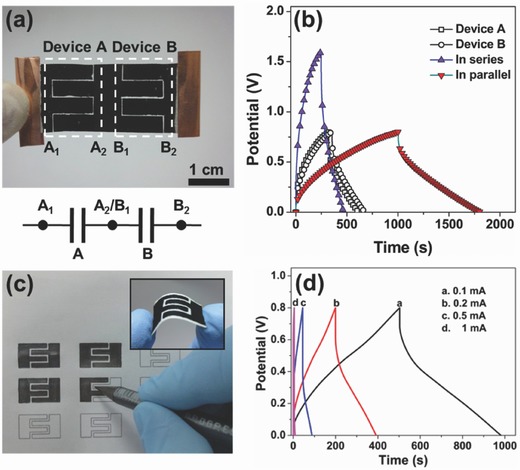
Flexible paper‐based interdigital solid‐state supercapacitors. a) Optical photograph of the as‐fabricated planar structured solid‐state supercapacitor with polypyrrole‐coated paper electrodes (polymerization time of 180 minutes). The corresponding schematic diagram is plotted below. Reproduced with permission.^12^ Copyright 2013, Royal Society of Chemistry. b) Galvanostatic charge–discharge curves for the as‐fabricated planar supercapacitor with different device configuration at a fixed current of 1 mA. c) Optical picture of using a pencil to draw a conductive graphite layer on the as‐printed interdigital patterns to obtain an interdigital substrate. The insert shows the flexible planar interdigital electrodes deposited polyaniline on the interdigital graphite substrate. d) Charge and discharge curves of the planar interdigital supercapacitor with G/PANI‐Paper electrodes at different currents. Reproduced with permission.^14^ Copyright 2013, Elsevier.

Paper‐based planar interdigital supercapacitors hold many advantages compared with traditional sandwiched structure, such as the improved ion diffusion and easy integration with other planar flexible electronics devices. Yuan et al. successfully fabricated two planar interdigital supercapacitors on one piece of PPy‐coated paper (**Figure** **17**a–b).^12^ The two supercapacitors (denoted as Devices A and Devices B) showed a planar structure, both with 2 pairs of interdigital‐shaped electrodes and coated with solid‐state PVA/H_3_PO_4_ electrolyte. By changing the linking between four terminals of A_1_, A_2_, B_1_ and B_2_, Devices A and Devices B exhibited a series or parallel relationship. Comparing the experimental capacitance and the calculated capacitance, it was found that these devices roughly obeyed the basic rule of series and parallel connections. Furthermore, Yao et al. proposed a novel method to fabricate paper‐based planar interdigital supercapacitors (Figure 17c–d).^14^ First, pencil was used to draw the as‐designed patterns on paper, forming a conductive interdigital graphite planar supercapacitor. Then PANI networks were electrodeposited on the interdigital electrodes of each side. The interdigital solid‐state planar supercapacitor device was fabricated by coating solid‐state PVA/H_2_SO_4_ electrolyte on the surface of the interdigital electrodes and the gaps in between. The symmetrical curves of charging and discharge parts displayed its good capacitive behavior.

### 4.2 Flexible Li‐Ion Batteries

Since 1991, the first commercial product released by Sony, Li‐ion batteries have attracted great interest due to their high energy density, high operation voltage, low self‐discharging rate, and relatively good long‐term stability.^10^ Recent increasing demand in flexible electronics and wearable electronics further drive the development of light‐weight, thin, and flexible Li‐ion batteries. However, currently most of the Li‐ion batteries are still adopting the conventional rigid, heavy and bulky configuration.^283^, ^284^ Here we will review the recent progress in making flexible paper‐based or paper‐like anodes and cathodes that are the key components for Li‐ion batteries.

Generally, anode and cathode will be fabricated by mixing powder of active materials with conducting agents such as carbon black and polymeric binders such as PTFE or PVDF to form slurry and then coated onto Cu foil and Al foil, respectively. The main drawback for this electrode configuration is that the active materials are likely to detach from the smooth conductive substrates under the bending states.^102^ To solve this problem, developing new electrodes with robust mechanical flexibility is necessary. Graphene and CNTs are two of the most important carbonaceous materials with excellent electrical conductivity. Paper‐like assembly with 3D porous structure of graphene flakelets or CNT wires with rough surface have been widely investigated in recent years. Yet, pure graphene and CNTs paper often suffer from large irreversible capacity, fast capacity fade, low capacities and safety issue due to Li dendrite formation between electrode and separator, resulting in short circuit.^128^, ^197^ Recent studies showed that hybridizing active electrode materials with graphene or CNTs to fabricate flexible and free‐standing graphene‐ or CNTs‐based electrodes are effective in addressing the problem.

**Figure 18:**
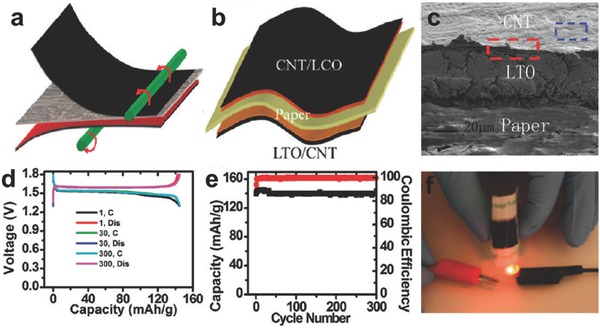
Flexible secondary Li‐ion paper batteries. a) Schematic of the lamination process: the freestanding film is laminated on paper with a rod and a thin layer of wet PVDF on paper. b) Schematic of the final paper Li‐ion battery device structure, with both LCO/CNT and LTO/CNT laminated on both sides of the paper substrate. The paper is used as both the separator and the substrate. c) SEM image of the cross section for the laminated paper Li‐ion battery. The layers thicknesses are CNT ≈2 µm, LTO ≈30 µm, paper ≈100 µm, LCO ≈30 µm, and CNT ≈2 µm. d) Galvanostatic charging/discharging curves of the LTO anode (1.3–1.7 V) half cells with conductive paper current collectors. The mass of the LTO electrode is 1.8 mg. The current rate is C/5. e) Cycling performance of LTO nanopowder (C/5, 0.063 mA) half cells. f) Flexible Li‐ion paper batteries light an LED device. Reproduced with permission.^15^ Copyright 2010, American Chemical Society.

Hu et al. employed a doctor blade method to coat CNT ink on stainless steel substrate to form a conducting layer, followed by the further blade‐coating of Li_4_Ti_5_O_12_ (LTO) and LiCoO_2_ (LCO) separately (**Figure** **18**).^15^ The LTO/CNT and LCO/CNT films were then easily delaminated from the substrates by simply immersing them into DI water and peeling off. After that, the LTO/CNT and LCO/CNT films were rolled to the paper via coating a thin layer of PVDF in between, which the PVDF functions as a glue to stick the double layer films on paper. This is the first demonstration using commercial paper in Li‐ion batteries, where paper is used as both separator and mechanical support. This Li‐ion paper battery is thin, about 300 µm in total, and yielded a discharging capacity of 120 mAh g^−1^. Besides, this device showed excellent self‐discharge performance that only 0.54 mV voltage drop was observed for this full cell after 350 hours.

Incorporation of materials with high theoretical capacity, such as Si (4200 mAh g^−1^),^285^ Ge (1600 mAh g^−1^),^286^ Sn (992 mAh g^−1^),^287^ Fe_2_O_3_ (1005 mAh g^−1^),^288^ Co_3_O_4_ (890 mAh g^−1^),^289^ MoO_2_ (838 mAh g^−1^),^290^ SnO_2_ (781 mAh g^−1^),^291^ CuO (674 mAh g^−1^),^292^ V_2_O_5_ (294 mAh g^−1^),^222^, ^293^ with graphene or CNTs to make free‐standing paper‐like electrodes have attracted considerable interest. Nanostructured active materials are uniformly distributed in these 3D conductive networks and could effectively reduce the polarization and pulverization associated with large volume change during lithiation and delithiation process.^163^, ^164^, ^294^

Zhou et al. reported a Fe_2_O_3_/SWCNTs paper with a ultrahigh Fe_2_O_3_ loading of 88 wt% prepared by oxidizing a floating catalyst chemical vapor deposition‐assembled Fe/SWCNT paper.^294^ This flexible paper showed a high reversible capacity of 1243 mAh g^−1^ at a current density of 50 mA g^−1^ and a good cyclic stability over 90 cycles. CNTs can also be used to fabricate MXene/CNT paper via vacuum filtration method. The porous Ti_3_C_2_T_x_/CNT paper electrode displayed a high capacity of about 1250 mAh g^−1^ at 0.1 C and good rate performance of 330 mA g^−1^ at 10 C. This excellent performance was attributed to the highly conductive 3D CNT network as well as the porous characteristic of 2D MXene nanoplate.^218^ Graphene can also be used in vacuum filtration to assemble flexible free‐standing paper‐like electrodes. Zhao et al. used acid‐sonication treatment to create graphene with carbon vacancies followed by filtration of these porous graphene with Si nanoparticle to make free‐standing Si/graphene paper. These paper electrodes achieved an outstanding reversible capacity of 3200 mAh g^−1^ at 1 A g^−1^ and a good rate capacity of 1100 mAh g^−1^ at 8 A g^−1^ as well as good cycling stability which repeated up to 99.9% between cycles for over 150 cycles.^163^

### 4.3 Flexible Li‐S Batteries

Lithium‐ion batteries have achieved a great success in the past three decades and have been applied for many flexible electronics devices. However, the state‐of‐the‐art Li‐ion batteries are still not able to completely satisfy the increasing demand for applications such as electric vehicles and grid‐level energy storage that require extremely high energy density for the emerging applications.^295^

Lithium‐sulfur (Li‐S) batteries are promising candidates for the next generation rechargeable batteries due to their higher theoretical specific capacity (1673 mAh g^−1^) and energy density (2500 Wh kg^−1^) compared with Li‐ion batteries.^22^, ^295^ The key difference between these two batteries is the charge storage mechanism. Li‐ion batteries store charges through the intercalation of lithium ion into layered active materials, such as graphite or layered metal oxides. Because the lithium ions can only be intercalated topotactically into some specific sites, their theoretical capacity and energy density are typically limited to 370 mAh g^−1^ and 420 Wh kg^−1^.^22^ In contrary, Li‐S batteries store charges through a multi‐electron electrochemical redox reaction (16*Li* + *S*_8_ → 8*Li*_2_ *S*) involving the metal plating and stripping on the lithium anode (*Li* ^+^ + *e* ^−^ → *Li*) and a conversion reaction on the sulfur cathode (*S*_8_ + 16*Li* ^+^ + 16*e* ^−^ = 8*Li*_2_ *S*). These reactions are non‐topotactic and therefore lithium anodes and sulfur cathodes have excellent theoretical specific capacities of 3860 and 1673 mAh g^−1^, respectively.^22^ Besides, the average of the lithium‐sulfur cell voltage of 2.15 V, they can achieve a theoretical energy density of 2500 Wh kg^−1^.

Li‐S batteries have been first reported in 1962.^296^ After decades of intensive research, such batteries are still plagued with low discharge capacity and fast capacity decay during cycling. In 2009, Nazar et al. reported a highly ordered nanostructured carbon‐sulfur cathode for Li‐S battery by encapsulating sulfur within the mesopores of mesoporous carbon CMK‐3, and demonstrated its high discharge capacity of 1320 mAh g^−1^ and stable cycling performance over 20 cycles.^16^ Since then, significant progress has been made in the field of Li‐S batteries.

The main problems impeding the application of Li‐S batteries include the low electrical conductivity of sulfur (5 × 10^−30^ S cm^−1^), the dissolution of intermediate lithium polysulfides, and the large volume expansion (80%) of sulfur during lithiation.^22^ To overcome these challenges, various materials are employed to immobilize S for enhancing the electrical conductivity of the S electrode and preventing the dissolution of polysulfides, including carbon materials, such as carbon nanotubes,^297^, ^298^ graphene,^299^, ^300^ carbon nanofibers,^301^, ^302^ porous carbon,^303^, ^304^ and hollow carbon spheres,^305^ conducting polymers like polyaniline (PANI),^306^ polypyrrole (PPy)^18^, ^307^ and poly(3,4‐ethylenedioxythiophne)‐ poly(styrenesulfonate) (PEDOT/PSS),^308^ metal oxides, such as TiO_2_,^309^ Ti_4_O_7_,^310^ MnO_2_,^311^ indium tin oxide (ITO),^312^ Fe_2_O_3_,^313^ La_2_O_3_,^314^ VO_2_,^315^ V_2_O_5_,^316^ MoO_3_,^316^ Nb_2_O_5_,^317^ and metal hydroxides including Ni(OH)_2_ 318 and Co(OH)_2_.^319^, ^320^ Among these host materials, graphene, CNTs and carbon fibers are proved to be very efficient in fabricating flexible Li‐S electrodes.^18^, ^157^, ^298^, ^302^

**Figure 19:**
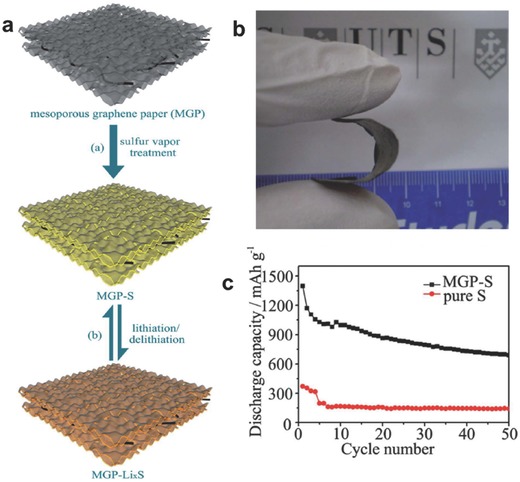
Flexible paper‐based Li‐S battery. a) A schematic illustration for the preparation of mesoporous graphene paper–sulfur electrodes and application as cathode in lithium–sulfur batteries. b,c) Digital photographs of the MGP paper, exhibiting an intact paper‐like morphology (b) and flexible property (c). Reproduced with permission.^157^ Copyright 2013, Royal Society of Chemistry.

Huang et al. prepared mesoporous graphene‐sulfur paper by filtration and sulfur vapor treatment approach (**Figure** **19**).^157^ Amorphous sulfur was homogeneously distributed and immobilized in the mesoporous architectures of porous graphene paper. The free‐standing, binder‐free as‐prepared mesoporous graphene‐sulfur papers can be directly used as electrodes in lithium‐sulfur batteries, which yielded a high specific discharge capacity of 1393 mAh g^−1^ and retain 689 mAh g^−1^ after 50 cycles. Li et al. demonstrated a foldable lithium‐sulfur battery by depositing sulfur on the checkerboard patterned super elastic CNT film.^298^ This folded lithium‐sulfur battery could be folded along two mutually orthogonal directions and showed <12% loss in specific capacity over 100 continuous folding and unfolding cycles. Such flexible and foldable lithium‐sulfur batteries show their potential in powering the flexible and foldable devices including laptops, cell phones, tablet computers, surgical tools and implantable biomedical devices in the future.

### 4.4 Flexible Li‐O_2_ Batteries

Lithium‐oxygen (Li‐O_2_) batteries are also one of the most promising energy storage systems and have attracted worldwide attention owing to its super‐large theoretical energy density.^17^ When an oxygen cathode is coupled with a lithium anode, according to the reaction 2*Li* + *O*_2_ → *Li*_2_ *O*_2_, which includes a cathodic reaction of 2*Li* ^+^ + *O*_2_ + 2*e* ^−^ → *Li*_2_ *O*_2_ and an anodic reaction of 2*Li* − 2*e* ^−^ → 2*Li* ^+^, which could achieve an energy density of 3600 Wh kg^−1^ [(capacity of 1200 Ah kg^−1^) × (operating voltage of 3.0 V)], which is nearly one order of magnitude larger than the conventional lithium‐ion batteries (420 Wh kg^−1^). The first Li‐O_2_ battery, with a structure of Li/organic electrolyte/air, was assembled and investigated in 1996.^321^ However, this type of device did not receive much attention until Bruce et al. demonstrated its rechargeable ability recently.^322^ Since the research on rechargeable Li‐O_2_ batteries is still in its infancy, it remains very challenging to fabricate a high performance Li‐O_2_ battery, so flexible or foldable Li‐O_2_ batteries have rarely been demonstrated. However, several groups have attempted to assemble flexible Li‐O_2_ batteries in the laboratory and some prototypes have been successfully developed.^19^, ^323^

**Figure 20:**
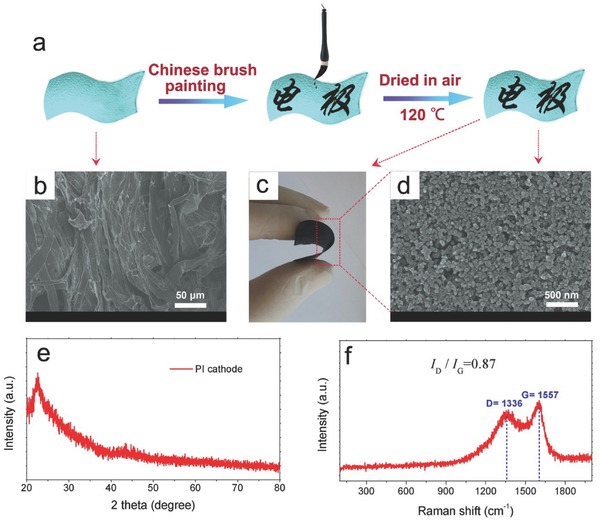
Flexible paper‐based Li‐O_2_ battery. a) Schematic representations for the design and preparation of the PI cathode. b) SEM image of pristine paper. c) Photograph of the obtained flexible PI cathode. d) Enlarged image of (c). e) XRD pattern and f) Raman spectrum of the obtained PI cathode. Reproduced with permission.^19^

Liu et al. adopted an ancient Chinese brush painting method to deposit the commercial carbon ink on commonly used paper (**Figure** **20**).^19^ This flexible low‐cost paper‐ink (PI) cathode can be directly applied in Li‐O_2_ batteries by combining with lithium belt anode, glass fiber separator and non‐aqueous electrolyte (1M lithium triflate in tretraethylene glycol dimethyl ether). The Li‐O_2_ battery showed a high capacity of about 6500 mAh g^−1^ at a current density of 200 mA g^−1^ and reached about 50 cycles with the capacity restricted to 1000 mAh g^−1^ at a current density of 200 mA g^−1^. The PI cathode after bending 1000 cycles did not show obvious deterioration in performance. Besides, the electrochemical performance of the Li‐O_2_ battery can be further improved via coating Ru nanoparticles on the PI cathode.

## 5 Conclusions and Outlook

The rapid development of flexible electrodes has open up new opportunities for flexible energy storage systems and wearable electronics. Among them, paper‐based electrodes as a new class of electrode configuration have attracted a lot attention. In this review, we have summarized the recent advances in this area, with a particular focus on the methodology of fabricating these novel functional electrodes, including paper‐supported electrodes and paper‐like electrodes. Finally, in this section, we will discuss some key challenges and opportunities in this fast growing field. First, paper‐based electrodes have been widely used in flexible energy storage devices such as supercapacitors and Li‐ion batteries. However, their application in Li‐S and Li‐O_2_ batteries, as well as some new types of energy storage system like Na‐ion batteries, Mg‐ion batteries has been rarely investigated. In exploring the potential use of paper‐based electrodes in these areas, some issues should be considered. For example, to use paper‐based electrodes for Li‐S batteries, the electrode is required to have not only good electrical conductivity and flexibility, but also strong affinity with sulfur. When they are used in Na‐ion batteries and Mg‐ion batteries, a critical problem would be whether these paper‐based electrodes is mechanically/structurally robust that can accommodate large volume change during Na‐ion and Mg‐ion insertion and desertion, since these ions are much larger than Li‐ions.

Second, we believe that performance standards should be established for paper‐based electrodes devices. For instance, in order to better evaluate the practicality of supercapacitor electrode, the capacitance/capacity of the paper‐based electrodes should be calculated based on the total mass/volume of the electrodes, including both electrochemically active materials and inactive cellulose paper substrates, instead of the mass or volume of the active materials. For paper‐like or film electrode, it is also critical to provide the areal capacitance/capacity of the electrode. Likewise, the energy density and power density of paper‐based supercapacitors should be normalized to the mass, areal or volume of the entire device, including electrodes, electrolyte, separator and packaging materials.

Third, paper‐based electrodes with high‐mass loading of active materials (e.g*.*, larger than 10 mg cm^−2^) should be explored for practical application. Currently, the mass loading of active materials for paper‐based electrodes are still in the range of several µg to several mg. This relatively low loading is difficult to meet the increasing demand for high energy density device for powering flexible electronics. Higher areal capacitance/capacity of paper‐based electrode can be realized by simply increasing the film thickness. The challenge is to retain efficient ion diffusion in a thick paper‐based electrode, and thus, achieving high areal capacitance at fast charging/discharging rate.

Fourth, electrochemical property and stability of paper‐based flexible energy storage devices under extreme conditions should be investigated as they are of great importance for some practical applications. Previous studies have primarily focused on the performance of paper‐based flexible energy storage devices under dry and ambient environment. Their performance under harsh environments, such as extremely humid conditions and high/low temperatures, is rarely reported.

Fifth, new materials and fabrication techniques should be explored for making novel paper‐based electrodes. For example, optically transparent paper‐based electrodes and flexible energy storage devices can be implemented into all‐transparent electronic devices. Self‐healing paper‐based electrodes can repair the damage within the electrodes and extend their lifespan, which can be critical for certain energy storage devices. Investigation on new materials as well as fabrication processes could open up new opportunities for flexible paper‐based energy storage devices.

Sixth, new electrolytes should be explored for flexible paper‐based energy storage devices. Electrolytes can significantly affect the specific capacitance/capacity, energy density, power density and the cycling stability of flexible energy storage devices. An ideal electrolyte used in flexible paper‐based energy storage devices should be highly flexible, non‐flammable, environmentally friendly and has a unique combination of properties such as high voltage window, high ionic conductivity, low self‐discharging rate and good affinity with electrode materials. The flexible electrolyte which can simultaneously satisfy these requires has rarely been reported.

Seventh, the mechanical flexibility and stability for flexible paper‐based energy storage devices need to be thoroughly investigated and quantified. To date, many paper‐based devices are claimed to be flexible, however, the meaning of “flexibility” in different flexible devices can be considerably different. The electrochemical performances of a flexible paper‐based device under different bending states (e.g., bending angle) should be provided. Additionally, the device stability as a function of bending cycles would be another important data for comparison.

Eighth, it is critical that paper‐based flexible energy storage devices can couple with other flexible devices. For example, paper‐based energy conversion and storage devices can be integrated to form a self‐powered paper‐based system that can be used in smart flexible electronic applications. Due to their unique low‐cost and environmental friendly nature, this kind of integral multifunctional paper‐based electronic devices can be used to make disposable paper devices.

Last, a better understanding on the interfacial interactions between paper‐based electrodes and electrolyte under mechanical deformation is critical for developing high performance flexible devices. Current research on flexible paper‐based energy storage devices is still under the stage of reporting the experimental observations, rather than investigating their mechanisms. Advanced computational techniques can be used to simulate the paper‐based electrode surface behavior under physical distortion and predict its effect on the electrical conductivity and electrochemical properties.

## Conflict of Interest

The authors declare no conflict of interest.
